# A shift in PKM2 oligomeric state instructs adipocyte inflammatory potential

**DOI:** 10.1172/jci.insight.185914

**Published:** 2025-11-24

**Authors:** Michelle S.M.A. Damen, Pablo C. Alarcon, Calvin C. Chan, Traci E. Stankiewicz, Hak Chung, Keisuke Sawada, Cassidy J. Ulanowicz, John Eom, Jarren R. Oates, Jennifer L. Wayland, Jessica R. Doll, Rajib Mukherjee, Miki Watanabe-Chailland, Lindsey Romick-Rosendale, Sara Szabo, Michael A. Helmrath, Joan Sanchez-Gurmaches, Maria E. Moreno-Fernandez, Senad Divanovic

**Affiliations:** 1 Department of Pediatrics, University of Cincinnati College of Medicine, Cincinnati, Ohio, USA.; 2 Division of Immunobiology, Cincinnati Children’s Hospital Medical Center, Cincinnati, Ohio, USA.; 3 Immunology Graduate Program, University of Cincinnati College of Medicine, Cincinnati, Ohio, USA.; 4 NMR Metabolomics Core, Cincinnati Children’s Hospital Medical Center, Cincinnati, Ohio, USA.; 5 Medical Scientist Training Program, University of Cincinnati College of Medicine, Cincinnati, Ohio, USA.; 6 Center for Stem Cell & Organoid Medicine (CuSTOM),; 7 Division of Endocrinology,; 8 Division of Developmental Biology,; 9 Division of Pathology,; 10 Division of Pediatric General and Thoracic Surgery, and; 11 Center for Inflammation and Tolerance, Cincinnati Children’s Hospital Medical Center, Cincinnati, Ohio, USA.

**Keywords:** Immunology, Inflammation, Metabolism, Adipose tissue, Cytokines, Obesity

## Abstract

Processes that promote white adipocyte inflammatory function remain incompletely defined. Here, we demonstrated that type I interferon–dependent (IFN-I–dependent) skewing of adipocyte glycolysis, nicotinamide adenine dinucleotide (NAD^+^) utilization, and pyruvate kinase isozyme M2 (PKM2) function may contribute to increased systemic and tissue inflammation and disease severity in obesity. Notably, chemical and/or genetic inhibition of glycolysis, the NAD^+^ salvage pathway, or PKM2 restricted IFN-I–dependent increase in adipocyte inflammatory cytokine production. Further, genetic or small molecule targeting of PKM2 function in vivo was sufficient to reduce systemic and tissue inflammation and metabolic disease severity in obese mice, in an adipocyte PKM2-dependent manner. Further, white adipose tissue of individuals living with obesity and metabolic disease, compared with metabolically healthy individuals with obesity, showed an increase in expression of inflammatory and metabolic genes, while small molecule targeting of PKM2 function contributed to reduced IFN-I–driven inflammatory cytokine production by primary human adipocytes. Together, our findings invoke the IFN-I/PKM2 axis as a potential target for modulating adipocyte dysregulated inflammation.

## Abstract 2

White adipocyte metabolism modulates their proinflammatory cytokine production. Altering a specific enzyme in the glycolytic pathway instructs this inflammation and potentially reduces obesity disease severity.

## Graphical abstract

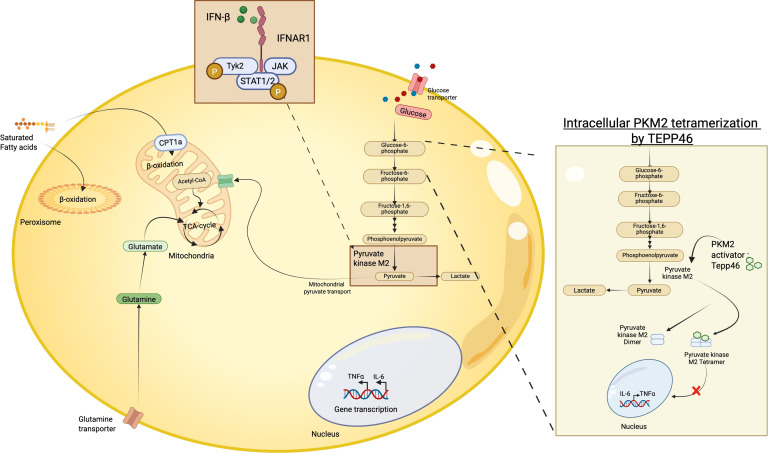

## Introduction

White adipocytes are traditionally known for their ability to store lipids (1). However, white adipocytes also possess immune-like properties as they express innate immune receptors, act as antigen-presenting cells, and produce various cytokines, chemokines, and adipokines (2, 3). Cumulatively, dysregulation of these immune-like functions in white adipocytes (1, 4) and/or white adipose tissue (WAT) is associated with increased disease pathogenesis of type II diabetes (1), cardiovascular disease (5), metabolic dysfunction–associated steatotic liver disease (6), inflammatory bowel disease (7), rheumatoid arthritis (8), and cancer (9). However, mechanisms that instruct white adipocyte inflammatory vigor remain poorly defined.

Obesity-driven alterations of WAT homeostasis promote immune cell infiltration and enhance systemic and tissue inflammation (10). In obesity, increased systemic levels of inflammatory mediators (e.g., endotoxin [LPS], DNA, free fatty acids) (11) activate TLR signaling cascades and amplify production of immune mediators including type I IFNs (IFN-Is) (12). The IFN-I family of cytokines, in mice and humans, consists of IFN-α (14 mouse and 13 human subtypes), IFN-β, IFN-ε, IFN-κ, IFN-ο, and IFN-ϖ (in humans) or limitin (in mice) (13, 14). IFN-Is are central to activation of both innate and adaptive immune responses and signal through the ubiquitously expressed interferon-α/β receptor (IFNAR). IFNAR signaling activates the canonical JAK/STAT pathway (15), subsequently increasing interferon regulatory factor and interferon stimulatory gene expression (16). IFN-I axis activation, including adipocyte-specific IFNAR signaling, contributes to severity of metabolic diseases in obesity (12). Notably, IFN-I sensing alters cellular metabolism in immune cells (17), while cellular metabolism instructs tissue homeostasis and immune cell function and cytokine production (18–22) — a concept recently expanded to adipocytes (e.g., IL-6, TNF) (17, 23, 24). In fact, glucose, amino acids, phosphocreatine, and insulin are proposed as key processes that link cellular metabolism and adipocyte inflammation (25–27).

To facilitate rapid cytokine production associated with inflammation, cells preferentially utilize glycolysis and tricarboxylic acid (TCA) cycle–driven oxidative phosphorylation to generate high levels of adenosine triphosphate (ATP) (28, 29). Increased generation of ATP is in part achieved through increased expression of pyruvate kinase isozyme M2 (PKM2; the final and rate-limiting step of glycolysis) (28). PKM2 can shift from the pyruvate kinase enzymatically inactive monomeric or dimeric state to the enzymatically active oligomeric/tetrameric state (30). The PKM2 shift to the oligomeric/tetrameric form drives the conversion of phosphoenolpyruvic acid (PEP) to pyruvate, supplying the TCA cycle with its main metabolite, acetyl coenzyme A (acetyl CoA), to maintain high production of ATP and high immunometabolic activity. In contrast, the preferential shift to the monomeric/dimeric state of PKM2, high in protein kinase activity, translocates to the nucleus to enhance gene transcription (31). Importantly, various posttranslational mechanisms (e.g., acetylation, succinylation, phosphorylation) are linked with preferential modification/skewing of PKM2 state (32–35). In addition, the glycolysis intermediate nicotinamide adenine dinucleotide (NAD^+^) modulates PKM2 oligomeric state and downstream effector functions through sirtuin 5 (SIRT5) (34).

Here, on the above-introduced scientific basis, we investigated whether IFN-I mediates changes in PKM2 oligomeric state and its downstream functions, impacts white adipocytes’ inflammatory cytokine production, and contributes to metabolic disease severity in obesity. We show that IFN-I sensing alters expression of both inflammatory and cellular metabolism–associated genes/pathways in primary mouse adipocytes. These findings correlate with functional skewing of glycolysis and changes in cofactor NAD^+^ levels and both ATP/ADP and ATP/AMP ratios in adipocytes. Blockade of the enzymatically active tetrameric PKM2 state by shikonin or TEPP-46 or blockade of NAD^+^ signaling by E-daporinad (nicotinamide phosphoribosyltransferase [Nampt] inhibitor) blunts adipocyte inflammatory cytokine production in an IFNAR-dependent manner in vitro. In contrast, exogenous administration of β-nicotinamide mononucleotide, a precursor for a required component of NAD^+^ production, enhances IFN-I–driven induction of white adipocyte pro-inflammatory cytokine production. Notably, treatment of obese WT mice with TEPP-46 or genetic deletion of PKM2 in adipocytes, using *Pkm2^fl/fl^ Adipoq^Cre+^* mice, restricts tissue inflammation and metabolic disease severity. Importantly, akin to mouse adipocytes, sensing of IFN-I by both primary and stromal vascular fraction–derived (SVF-derived) human white adipocytes was linked to exacerbated glycolysis and PKM2-dependent inflammatory cytokine production. Further, inhibition of adipocyte glycolysis and PKM2 function showed a stronger impact on pro-inflammatory cytokine production in white adipocytes from individuals living with obesity and metabolic disease compared with metabolically healthy individuals living with obesity. Together, these data suggest that the IFN-I/PKM2 axis may regulate white adipocyte inflammatory cytokine production and metabolic disease severity in obesity.

## Results

### IFN-I sensing unlocks white adipocyte inflammatory function.

IFN-I (12) and LPS levels (36) and adipocyte size and number are increased in obesity. Adipocytes respond to various IFN-Is (e.g., IFN-α and IFN-β) and LPS (12), with combined IFN-I and LPS sensing shown to additively increase adipocyte inflammatory cytokine production (12). However, whether such effects are conserved in males and females and are maintained across diverse adipocyte sources in mice is not well defined. To answer these foundational questions, we established a reductionist model that mimics IFN-I levels (recombinant IFN-β; a representative of IFN-Is) and/or increased endotoxemia (purified, TLR4 specific, LPS; mimic of endotoxemia) in an obese microenvironment (12). SVF-derived adipocytes from inguinal WAT (iWAT) from male or female WT mice were either mock-stimulated or stimulated with IFN-I or LPS alone or a combination of IFN-I + LPS. Unlike IFN-I stimulation, which did not alter cytokine production (data not shown [DNS] and ref. 12), LPS stimulation alone was sufficient to robustly induce pro-inflammatory cytokine production by adipocytes. Notably, combined sensing of IFN-I + LPS additively enhanced adipocyte inflammatory cytokine production in male (Supplemental Figure 1A; supplemental material available online with this article; https://doi.org/10.1172/jci.insight.185914DS1) and female (Supplemental Figure 1B) mice and correlated with increased expression of IFN-I axis genes (e.g., *Isg15*, *Oas1a*) in male (Supplemental Figure 1, C and D) and female mice (DNS). Further, these effects were not limited to SVF-derived white adipocytes, as primary mature white adipocytes isolated from epididymal WAT (eWAT) of WT male mice responded in a similar fashion (Supplemental Figure 1E). Together, these data demonstrate that combined sensing of IFN-I + LPS triggers enhanced cytokine production in white adipocytes and that such effects are conserved in both sexes and across adipocyte sources in mice. Given these foundational findings, for simplicity reasons, all subsequent studies focused on primary and/or SVF-derived white adipocytes from male mice. Further, the depiction of percentage change in cytokine production was used to allow for comparison among independently conducted studies.

How IFN-I + LPS sensing additively amplifies white adipocyte pro-inflammatory cytokine production is not understood. IFN-Is are sensed via ubiquitously expressed IFN-I receptor (IFNAR), while LPS is sensed via TLR4. Whether IFN-I or LPS sensing modulates IFNAR and TLR4 expression to in turn augment cytokine production is not known. Notably, combined sensing of these mediators, compared with LPS alone, did not alter *Ifnar* but decreased *Tlr4* gene expression in white adipocytes (Supplemental Figure 1, F and G). Thus, the contribution of IFNAR signaling to IFN-I + LPS sensing was examined next. In contrast with WT, SVF-derived, white adipocytes, adipocytes lacking IFNAR expression (IFNAR KO) failed to exhibit amplified inflammatory cytokine production following IFN-I + LPS stimulation (Supplemental Figure 1H). As various cytokines can additively amplify cytokine production in immune cells (37, 38) whether IFN-Is, among other cytokines, are somewhat unique and/or dominant in their ability to instruct white adipocyte cytokine production was examined next. Intriguingly, divergent from IFN-I observed effects, sensing of various pro- or antiinflammatory cytokines (IFN-γ, IL-12, TNF, IL-4, IL-10) combined with LPS failed to amplify cytokine production by adipocytes (Supplemental Figure 1I). These unexpected findings uncover a potentially unique ability of the IFN-I/IFNAR axis to regulate cytokine production in response to a secondary inflammatory stimulus (e.g., LPS) in white adipocytes.

**Figure 1:**
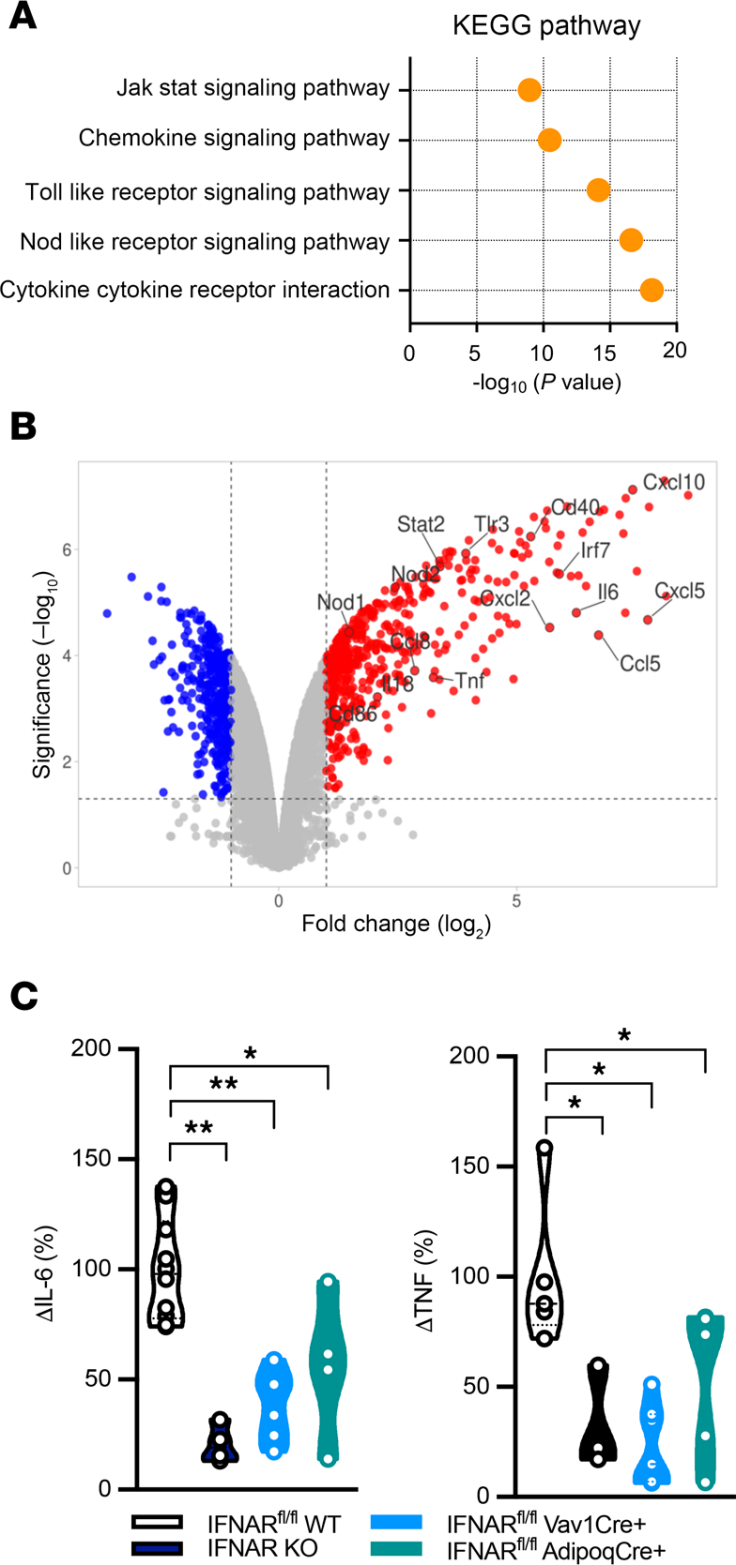
IFN-I/IFNAR axis modifies adipocyte transcriptome and inflammatory output. (**A** and **B**) WT mouse (SVF-derived) adipocytes were stimulated with vehicle control (saline) or recombinant IFN-β (rIFNβ) (250 U) for 3 hours and subsequently challenged with LPS (100 ng/mL) or not for 4 hours. (**A**) ToppGene pathway enrichment analysis of upregulated differentially expressed genes (fold change > 1.5; *P* value < 0.05) in rIFNβ + LPS–stimulated compared with vehicle-stimulated controls. Pathways related to inflammation are highlighted. KEGG, Kyoto Encyclopedia of Genes and Genomes. (**B**) Volcano plot of gene expression levels between rIFNβ + LPS–stimulated and vehicle stimulated adipocytes. Genes significantly upregulated in rIFNβ + LPS–stimulated adipocytes (fold change > 2, *P* value < 0.05) are represented by red dots. Genes significantly downregulated in rIFNβ + LPS–stimulated adipocytes (fold change < –2, *P* value < 0.05) are represented by blue dots. Upregulated genes related to inflammation depicted in **A** are highlighted. (**C**) IFNAR^fl/fl^, IFNAR-KO, IFNAR^fl/fl^ Vav1-Cre^+^, and IFNAR^fl/fl^ Adipoq-Cre^+^ mice were injected with biotinylated anti–IL-6 and anti-TNF antibodies (0.5 μg/mice), and after 3 hours mice were vehicle (saline)- or LPS (25 μg/mouse) -challenged via i.p. injections. Four hours later serum was collected, and IL-6 and TNF levels were measured via in vivo cytokine capture assay (IVCCA) ELISA. Data depict percentage change of cytokine levels in LPS-challenged mice compared with vehicle-challenged controls. A representative of 4–9 biological replicates. In violin plots, data present mean ± SEM. One-way ANOVA. *: *P* < 0.01; **: *P* < 0.001.

The signaling mechanisms behind IFN-Is’ ability to amplify white adipocyte cytokine production are poorly defined. RNA-Seq analysis of white adipocyte gene expression following IFN-I, LPS, and combined IFN-I + LPS treatment revealed that white adipocytes stimulated with IFN-I + LPS exhibited increased expression of inflammatory pathways, including JAK/STAT and TLR, compared with vehicle-stimulated controls (Figure 1A and Supplemental Figure 2, A–C). Such changes correlated with increased expression of various inflammatory genes known to contribute to pathogenic inflammation and disease severity in obesity, including *Tnf*, *Il-6*, *Cxcl10*, and *Nod1* (Figure 1B). Of note, the sensing of IFN-I alone or LPS alone increased expression of abovementioned inflammatory pathways above the unstimulated controls (Supplemental Figure 2, A and B). Importantly, the combined IFN-I and LPS stimulation was sufficient to further enhance the expression of abovementioned inflammatory pathways above the threshold observed in the LPS alone–treated group (Supplemental Figure 2C). Thus, the potential contribution of adipocyte IFNAR expression for systemic cytokine production was examined next. Mice with full-body IFNAR deletion (IFNAR KO), immune cell–specific IFNAR deletion (Vav1-Cre), or adipocyte-specific IFNAR deletion (Adipoq-Cre) and WT controls were challenged with LPS in vivo. As expected, whole-body IFNAR deletion yielded the most pronounced reduction in systemic inflammatory cytokine levels after LPS challenge. Surprisingly, however, in this setting, IFNAR deletion in adipocytes and IFNAR deletion in immune cells showed similar impact on systemic cytokine levels (Figure 1C). Together, these data suggest that adipocyte IFNAR signaling may contribute to both adipocyte and systemic inflammation.

### IFN-I sensing skews glycolytic pathway utilization in white adipocytes.

**Figure 2:**
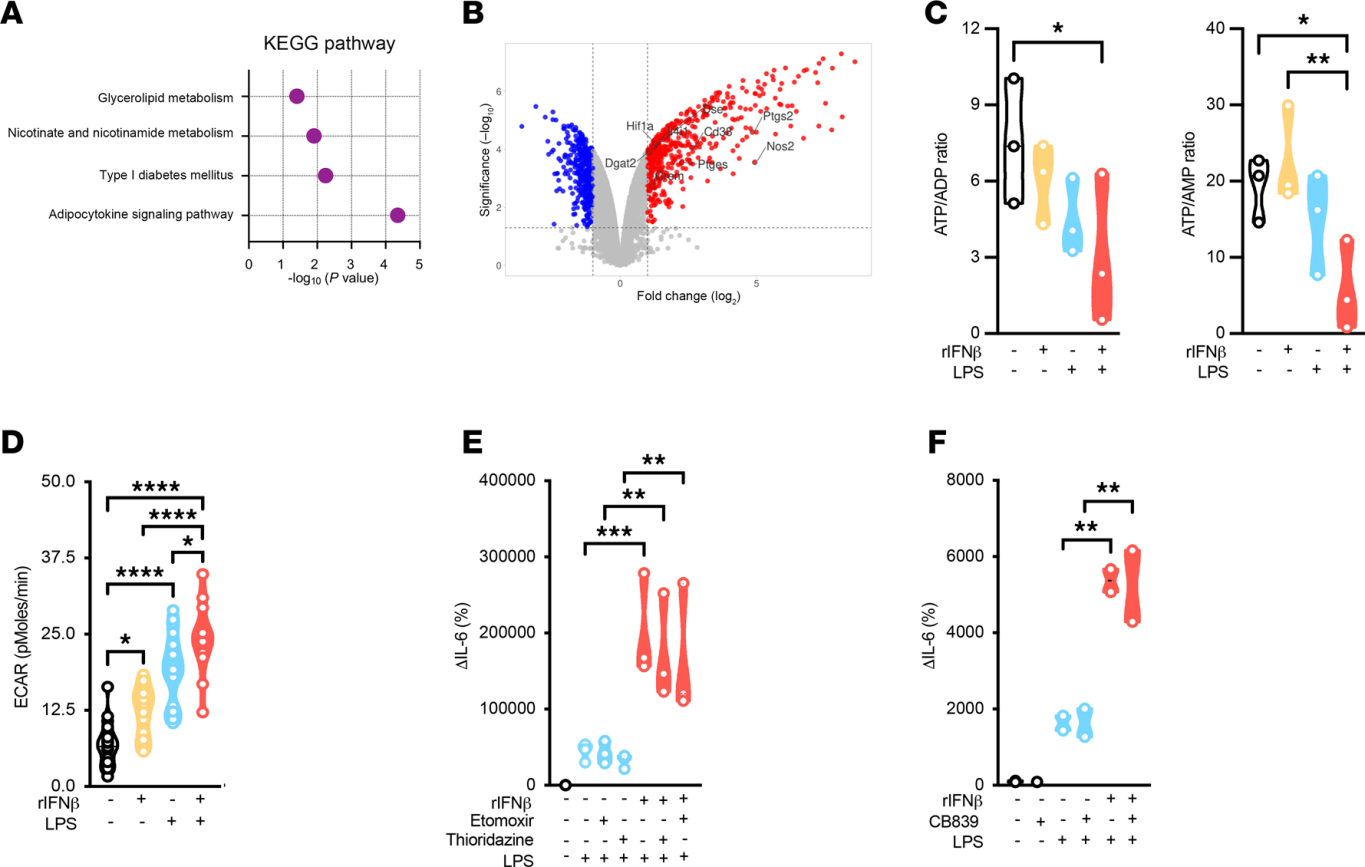
IFN-Is alter cellular glycolysis to amplify adipocyte inflammatory vigor. WT mouse (SVF-derived) adipocytes were stimulated with vehicle control (saline) or rIFNβ (250 U) for 3 hours and subsequently challenged with LPS (100 ng/mL) or not for 4 hours. (**A**) ToppGene pathway enrichment analysis of upregulated differentially expressed genes (fold change > 1.5; *P* value < 0.05) in rIFNβ + LPS–stimulated compared with vehicle-stimulated controls. Pathways related to cellular metabolism are highlighted. (**B**) Volcano plot of gene expression levels between rIFNβ + LPS–stimulated and vehicle-stimulated adipocytes. Genes significantly upregulated in rIFNβ + LPS–stimulated adipocytes (fold change > 2, *P* value < 0.05) are represented by red dots. Genes significantly downregulated in rIFNβ + LPS–stimulated adipocytes (fold change < –2, *P* value < 0.05) are represented by blue dots. Representative upregulated genes related to cellular metabolism depicted in **A** are highlighted. (**C**) ATP/ADP (left) and ATP/AMP ratio (right) in WT SVF-derived adipocytes upon vehicle, rIFNβ, LPS, or rIFNβ + LPS stimulation, quantified via NMR. (**D**) Extracellular acidification rate (ECAR) in WT SVF-derived adipocytes upon vehicle-, rIFNβ, LPS, or rIFNβ + LPS stimulation, quantified via Seahorse. (**E** and **F**) WT SVF-derived adipocytes were vehicle-, LPS-, or rIFNβ + LPS–stimulated in the presence or absence of thioridazine (peroxisomal fatty acid β-oxidation inhibitor; 10 μg/mL) and/or etomoxir (mitochondrial fatty acid β-oxidation inhibitor; 10 μg/mL) (**E**), or CB839 (glutaminolysis inhibitor; 10 μg/mL) (**F**). Culture supernatants were collected, and IL-6 levels were measured via ELISA. Data depict percentage change of IL-6 levels compared with vehicle-stimulated controls. A representative of 2–3 biological replicates. (**C**–**F**) In violin plots, data represent mean ± SEM. One-way ANOVA. *: *P* < 0.05; **: *P* < 0.001; ****: *P* < 0.00001.

IFN-I sensing skews cellular metabolism in immune cells (39, 40) and white adipocytes (12). Cellular metabolism shapes immune cell and adipocyte function (25, 27, 41–43). Nevertheless, the specific metabolic pathways impacted by IFN-I in white adipocytes remain unknown. RNA-Seq analysis of SVF-derived white adipocytes stimulated with IFN-I alone, LPS alone, and combined IFN-I + LPS, compared with mock-stimulated control, revealed enrichment of cellular metabolic pathways, including nicotinate and nicotinamide metabolism, in IFN-I + LPS combined conditions (Figure 2A and Supplemental Figure 3, A–C). Such changes correlated with increased expression of various metabolic genes that promote pathogenic inflammation and disease severity in obesity, including *Hif1a*, *Cd38*, and *Dgat2* (Figure 2B). Thus, whether these changes were sufficient to alter intracellular metabolite levels in white adipocytes was examined next. NMR spectrometric analysis revealed that IFN-I sensing alone caused a decrease in ATP/ADP ratios with no changes in ATP/AMP ratios. Yet, both ratios were additively restricted when IFN-I + LPS were sensed together (Figure 2C). Further, these changes in key components of cellular energetics were correlated with functional skewing of SVF-derived white adipocytes with sensing of either IFN-I or LPS alone yielding an increase in extracellular acidification rate (ECAR), which was additively amplified when IFN-I + LPS were sensed together (Figure 2D).

Inhibition of the entire glycolytic pathway using 2-deoxy-d-glucose (2-DG; a glucose analog that binds to hexokinase and blocks its function) decreased IFN-I–driven white adipocyte oxygen consumption rate and restricted IFN-I–driven increase in cytokine production by adipocytes (12). Whether these effects are unique to glycolysis or other cellular metabolic pathways could similarly impact white adipocyte function was examined next. In contrast with 2-DG, pharmacological inhibition of fatty acid oxidation (using etomoxir or thioridazine; Figure 2E) or glutaminolysis (using CB839; Figure 2F) failed to restrict IFN-I + LPS sensing–driven amplification of cytokine production by adipocytes. These data verify the importance of the glycolytic pathway in white adipocyte function and potentially suggest that IFN-I–dependent skewing of the glycolytic pathway activity may represent an important regulatory node of white adipocyte inflammatory function.

### IFN-I sensing skews PKM2 oligomerization in white adipocytes.

**Figure 3:**
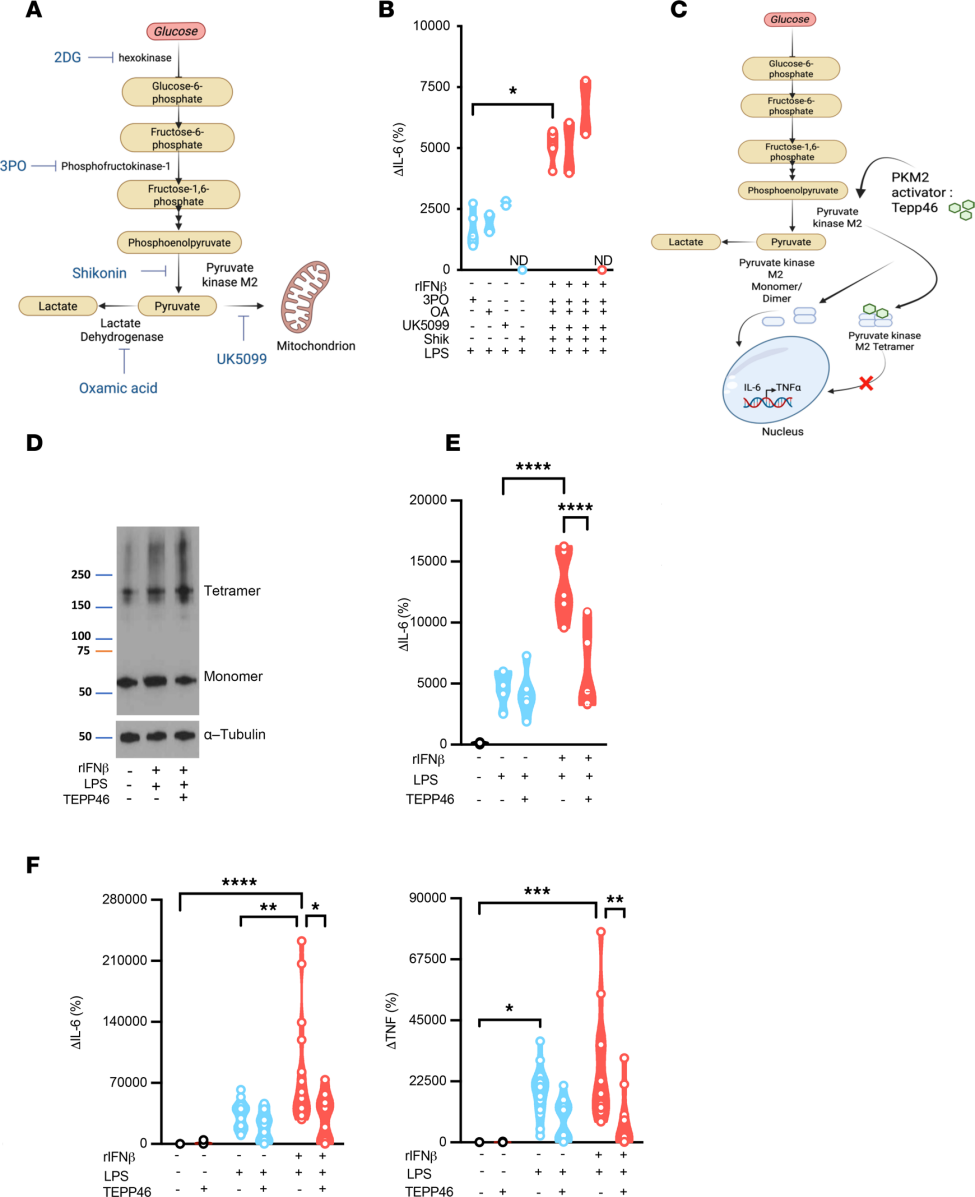
IFN-I sensing alters PKM2 function to instruct adipocyte inflammatory vigor. WT mouse (SVF-derived) adipocytes were stimulated with vehicle control (saline) or rIFNβ (250 U) for 3 hours and subsequently challenged with LPS (100 ng/mL) or not for 4 hours. (**A**) Schematic overview of glycolytic pathway with chemical inhibitors targeting different enzymatic steps in glycolysis. (**B**) WT SVF-derived adipocytes were vehicle-, LPS-, or rIFNβ + LPS–stimulated in the presence or absence of glycolytic metabolic inhibitors (3PO: 30 μM; oxamic acid: 10 μM; UK5099: 10 μM; shikonin: 10 μM). Culture supernatants were collected, and IL-6 levels were measured via ELISA. Data depict percentage change of IL-6 levels compared with vehicle-stimulated controls. Representative of 2 technical replicates and 3–5 biological replicates. (**C**) Schematic overview of TEPP-46 activity in glycolysis and cytokine production. (**D** and **E**) WT mouse (SVF-derived) adipocytes were vehicle-, LPS-, or rIFNβ + LPS–stimulated in the presence or absence of TEPP-46 (100 μM). (**D**) Protein expression of different configurations (monomer and tetramer) of PKM2 and α-tubulin (loading control) analyzed via Western blot. Representative of 2 independent experiments. (**E**) Culture supernatants were collected, and IL-6 levels were measured via ELISA. Data depict percentage change of IL-6 levels compared with vehicle-stimulated controls. (**F**) WT mice were twice i.p. injected with TEPP-46 (50 mg/kg/mouse; i.p.; daily), and subsequently i.p. injected with biotinylated anti–IL-6 and anti-TNF antibodies (0.5 μg/mice), with or without rIFNβ (10^4^ U/mouse). After 3 hours, mice were challenged or not with LPS (25 μg/mouse) via i.p. injections. Four hours later serum was collected, and IL-6 (left) and TNF levels (right) were measured via IVCCA ELISA. Data depict percentage change of cytokine levels in TEPP-46–treated compared with untreated controls. (**B**, **E**, and **F**) In violin plots, data represent mean ± SEM. One-way ANOVA. *: *P* < 0.05; **: *P* < 0.001; ***: *P* < 0.0001; ****: *P* < 0.00001.

Glycolytic activity is responsible for converting glucose to pyruvate and providing intermediates for the activation of additional metabolic pathways — a process dependent on multiple enzymes (44). However, the contribution of specific enzymes within the glycolytic pathway (Figure 3A) to white adipocyte ability to produce inflammatory cytokines is unknown. Interference of the glycolytic pathway using specific chemical inhibitors for phosphofructokinase (PFK) (using 3PO), lactate dehydrogenase (LDH) (using oxamic acid, [OA]), or mitochondrial pyruvate transport (using UK5099) failed to restrict IFN-I + LPS–driven amplification of white adipocyte inflammatory cytokine production (Figure 3B). In contrast, chemical inhibition of PKM2 (using shikonin) was sufficient to abrogate LPS alone– and IFN-I + LPS–driven amplification of adipocyte inflammatory cytokine production (Figure 3B). These findings suggest that PKM2 function may represent a critical step within the glycolytic pathway that regulates IFN-I–dependent amplification of white adipocyte inflammatory function.

The existence of various PKM2 states (e.g., tetramer, dimer, monomer) with divergent downstream effector functions makes PKM2 a favorable target for manipulation of cellular metabolism. Shifts in PKM2 state that favor a high protein kinase activity (monomer/dimer) over high pyruvate kinase activity (tetramer) formation increase inflammatory gene transcription and activate inflammatory cascades (45, 46). Notably, the ratio between monomeric/dimeric versus tetrameric state determines the direction of downstream effector functions of PKM2 within the cell. Currently, 2 distinct chemical modulators of PKM2, shikonin and TEPP-46, exist. Shikonin, a less specific modulator, alters multiple aspects of the glycolytic pathway, including the inhibition of PKM2 phosphorylation (necessary for PKM2 nuclear translocation), hexokinase 2 (the first step of glycolysis), and cellular glucose uptake via actions on glucose transporter 1 (the primary glucose uptake transporter) (47). In contrast, TEPP-46 is a specific PKM2 activator that regulates PKM2 oligomeric state and immune cell inflammatory output (45, 48). Specifically, TEPP-46 binds in between 2 subunits of PKM2 dimers to force the formation of the tetrameric state, subsequently reducing PKM2 monomer/dimer formation (Figure 3C). Hence, the impact of TEPP-46 on IFN-I–driven amplification of cytokine production in white adipocytes was examined next. Sensing of IFN-I + LPS by SVF-derived white adipocytes increased total PKM2 protein level by 1.5-fold (Figure 3D and Supplemental Figure 4A) and total monomeric form of PKM2 protein by 1.4-fold (Figure 3D and Supplemental Figure 4B), which correlated with a shift in PKM2 tetramer/monomer ratio by 1.2-fold (Figure 3D and Supplemental Figure 4C). Notably, treatment of adipocytes with TEPP-46 led to a slight decrease in total PKM2 protein level (a change by 0.8-fold) (Figure 3D and Supplemental Figure 3D), an expected reduction in PKM2 monomeric form by almost 2-fold (Figure 3D and Supplemental Figure 4E), and a positively correlation with a 1.8-fold increase in ratio of PKM2 tetrameric to monomeric form (Figure 3D and Supplemental Figure 4F).

The functional consequences of TEPP-46–driven skewing of PKM2 state in white adipocytes were examined next. SVF-derived white adipocytes were either mock-stimulated or stimulated with LPS or IFN-I + LPS, with or without TEPP-46. Addition of TEPP-46 alone or not TEPP-46 to LPS-stimulated white adipocytes had no impact on adipocyte cytokine production. However, the presence of TEPP-46 in the IFN-I + LPS–stimulated white adipocytes robustly restricted adipocyte cytokine production (Figure 3E). To examine the relevance of such findings in vivo, WT mice were mock-treated or treated with LPS or IFN-I + LPS, with or without TEPP-46, and systemic cytokine levels were quantified. Importantly, treatment with TEPP-46 in vivo robustly restricted systemic levels of inflammatory cytokines only in mice that sensed IFN-I + LPS (Figure 3F). Together, these findings link IFN-I–driven amplification of white adipocyte cytokine production with PKM2 function.

### IFN-I sensing skews NAD^+^ signaling to instruct adipocyte cytokine production.

**Figure 4:**
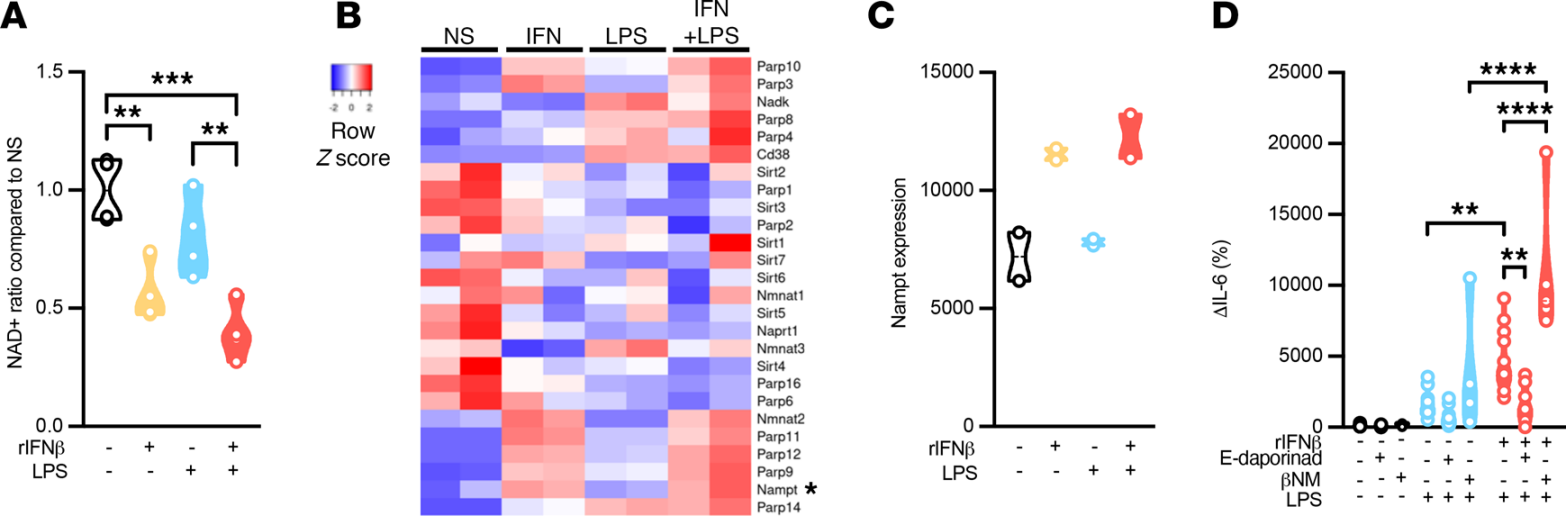
IFN-I sensing via skewed NAD^+^ utilization instructs adipocyte inflammatory vigor. WT mouse (SVF-derived) adipocytes were stimulated with vehicle control (saline) or rIFNβ (250 U) for 3 hours and subsequently challenged with LPS (100 ng/mL) or not for 4 hours. (**A**) Relative levels of NAD^+^ in rIFNβ-, LPS-, or rIFNβ + LPS–stimulated WT adipocytes compared with vehicle-stimulated controls, quantified by NMR. (**B**) Heatmap of NAD^+^ signaling–related genes measured via bulk RNA-Seq. (**C**) Nampt gene expression measured via bulk RNA-Seq. (**D**) WT mouse (SVF-derived) adipocytes were vehicle-, LPS-, or rIFNβ + LPS–stimulated in the presence or absence of E-daporinad (50 μM) or β-nicotinamide mononucleotide (10 μM). Culture supernatants were collected, and IL-6 levels were measured via ELISA. Data depict percentage change of IL-6 levels compared with vehicle-stimulated controls. (**A**, **C**, and **D**) In violin plots, data present mean + SEM. One-way ANOVA. *: *P* < 0.05; **: *P* < 0.001; ***: *P* < 0.0001; ****: *P* < 0.00001.

Cellular processes that instruct IFN-I–driven modification of glycolysis and PKM2 function remain poorly defined. Glycolytic activity requires utilization of NAD^+^ (a coenzyme critical for cellular metabolic function) to serve as an electron carrier for effective cellular glycolysis. Whether IFN-I sensing impacts NAD^+^ levels in white adipocytes was examined next. SVF-derived white adipocytes were either mock-stimulated or exposed to IFN-I alone, to LPS alone, or to IFN-I + LPS, and NAD^+^ levels were quantified. Whereas sensing of IFN-I or LPS alone reduced NAD^+^ levels, sensing of IFN-I + LPS resulted in additive reduction in NAD^+^ levels (Figure 4A). Such changes correlated with altered gene expression profiles associated with the NAD^+^ signaling cascade (Figure 4B). The concentration of NAD^+^ is a balance between NAD^+^ utilization and NAD^+^ synthesis via various pathways: the de novo biosynthetic pathway, the Preiss–Handler pathway, and the salvage pathway (49). In a setting of limited de novo NAD^+^ synthesis, the salvage pathway is the main source of NAD^+^ in maintaining cellular NAD^+^ levels. NAMPT is the rate-limiting enzyme in the NAD^+^ salvage pathway. Hence, the ability of IFN-I + LPS sensing to modify NAMPT expression in white adipocytes was examined next. Notably, unlike LPS sensing alone, both IFN-I alone or combined IFN-I + LPS sensing increased NAMPT expression in white adipocytes (Figure 4C). These findings invoke a potential link between IFN-I sensing and the NAD^+^ pathway.

Whether NAD^+^ signaling contributes to IFN-I + LPS sensing–driven amplification of white adipocyte cytokine production was examined next. SVF-derived white adipocytes from WT mice were either mock-stimulated or stimulated with IFN-I alone, LPS alone, or IFN-I + LPS, with or without NAMPT inhibitor (E-daporinad, MedChemExpress [MCE]; HY-50876). Whereas addition of E-daporinad alone or to LPS-stimulated white adipocytes failed to alter adipocyte cytokine production, presence of E-daporinad in combined IFN-I + LPS–stimulated white adipocytes significantly restricted adipocyte cytokine production (Figure 4D). In contrast, supplementing adipocyte cultures with an initiator of the NAMPT reaction, a key NAD^+^ intermediate (β-nicotinamide mononucleotide MCE; HY-F0004 10 μM), trended toward enhancement of adipocyte cytokine production after combined IFN-I + LPS sensing (Figure 4D). These findings invoke a potential link among IFN-I/NAD^+^/glycolysis axes in regulation of white adipocyte inflammatory cytokine production.

### Adipocyte PKM2 function may contribute to metabolic disease severity in obesity.

Adipocyte inflammation promotes severity of metabolic diseases in obesity. Whether PKM2 transcriptional activity impacts metabolic disease severity in vivo was examined next using both genetic deletion and chemical manipulation. To control for potential cre transgene– and microbiome-associated (50) off-target effects, WT (C57BL/6, nonlittermate), Pkm2^fl/fl^ Adipoq^Cre–^, and Pkm2^fl/fl^ Adipoq^Cre+^ (latter littermates) mice were compared at baseline. Chow diet (CD) feeding regimen, irrespective of mouse genotype, did not affect body weight, adiposity, spleen weight, and liver weight (Supplemental Figure 5). These data suggested unlikely adverse contribution of cre transgene or microbiome impact in this system at baseline and invoked the possibility that exogenous changes in adipocyte size/function (e.g., obesity) might be required to uncover the role of PKM2 in adipocytes. Next, to control for potential *loxP* transgene– (51), age-, microbiome-, and treatment-associated off-target effects, nonlittermate WT and Pkm2^fl/fl^ Adipoq^Cre–^ mice, untreated or vehicle control treated (DMSO; i.p. injection every 3 days for 2 weeks), were compared in the context of obesity. High-fat diet–fed (HFD-fed) WT and Pkm2^fl/fl^ Adipoq^Cre–^ mice, irrespective of genotype or DMSO treatment, exhibited similar diet-driven changes in total body weight, adiposity, spleen weight, liver weight, liver, systemic alanine aminotransferase (ALT) levels (marker of hepatocellular damage), and fasting glucose levels (Supplemental Figure 6). These data suggested that transgene, microbiome, and i.p. injection effects are unlikely to impact the outcomes of our in vivo studies. As such, to maximize the utility and reduce the number of control mice in obesity-related studies, data obtained from WT and Pkm2^fl/fl^ Adipoq^Cre–^ (untreated and vehicle treated) mice were combined (termed Pkm2^fl/fl^ Adipoq sufficient) and compared with data from Pkm2^fl/fl^ Adipoq^Cre+^ mice (termed Pkm2^fl/fl^ Adipoq deficient) in our subsequent studies.

**Figure 5:**
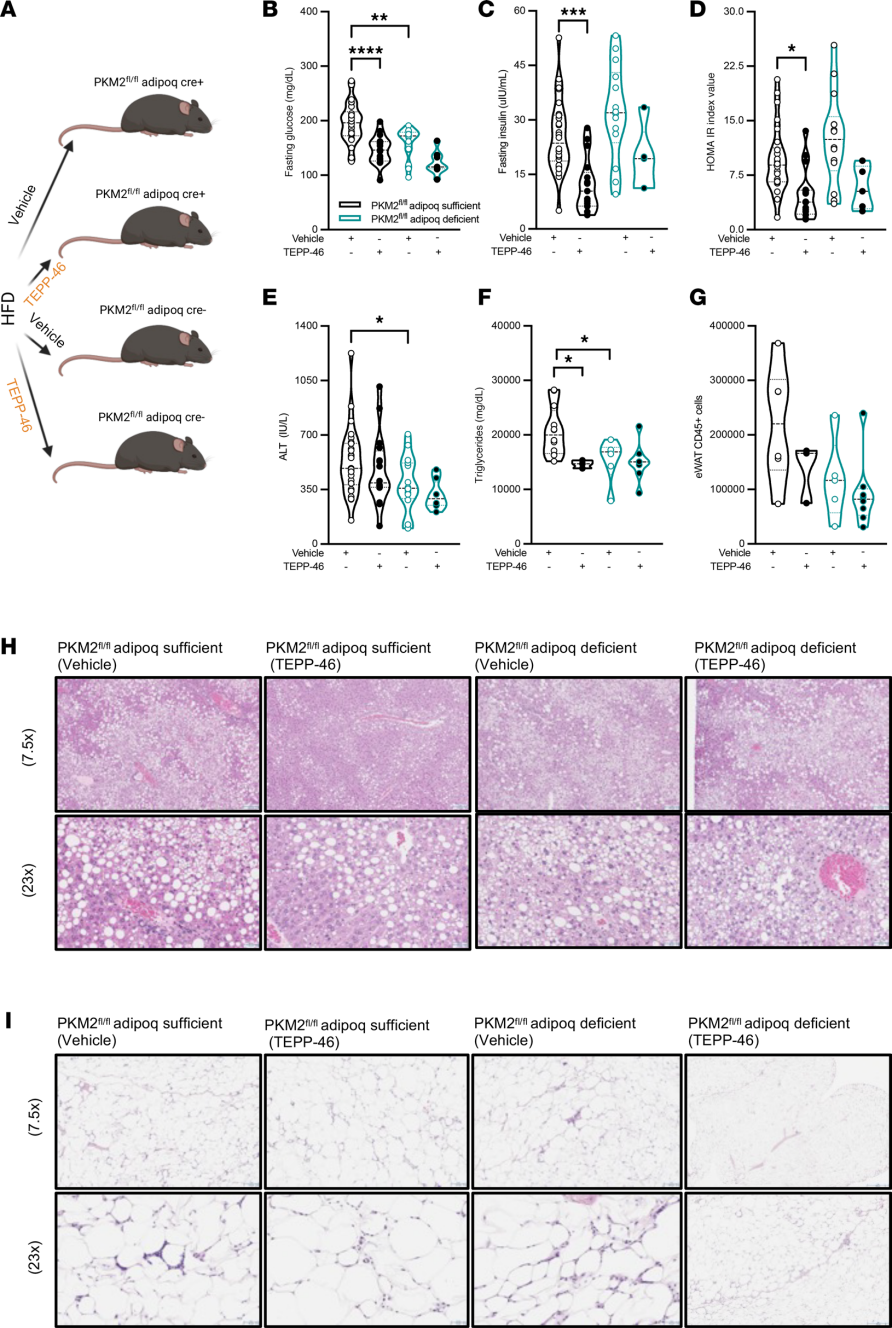
Improvement of metabolic disease severity following small molecule targeting of PKM2 function is in part dependent on adipocyte PKM2 expression. Adipocyte PKM2-sufficient (WT + PKM2^fl/fl^-Adipoq^Cre–^) and adipocyte PKM2-deficient (PKM2^fl/fl^-Adipoq^Cre+^) mice were fed HFD. After 34 weeks, mice were treated with vehicle control (DMSO) or TEPP-46 (37.5 mg/kg; i.p.) every 3 days for 2 weeks. (**A**) Schematic overview. (**B**) Fasting glucose. (**C**) Fasting insulin. (**D**) HOMA IR index values calculated using fasting glucose and fasting insulin levels. (**E**) Serum ALT levels. (**F**) Hepatic triglyceride levels. (**G**) Absolute number of CD45^+^ cells in eWAT, quantified by flow cytometry. (**H**) Representative H&E histology of liver taken at 7.5× (top row; scale bar = 137 μm) and 23× (bottom rows; scale bar = 45 μm). (**I**) Representative H&E histology of eWAT taken at 7.5× (top row; scale bar = 137 μm) and 23× (bottom rows; scale bar = 45 μm). (**B**–**G**) In violin plots, data present mean ± SEM. One-way ANOVA. *: *P* < 0.05; **: *P* < 0.001; ***P < 0.0001; ****: *P* < 0.00001.

First, the impact of genetic manipulation of adipocyte PKM2 on development of obesity and metabolic diseases was examined. HFD-fed Pkm2^fl/fl^ Adipoq sufficient (WT + Pkm2^fl/fl^ Adipoq^Cre–^) and Pkm2^fl/fl^ Adipoq deficient (Pkm2^fl/fl^ Adipoq^Cre+^) vehicle-treated mice (Figure 5A) exhibited similar total body weight, adiposity, spleen weight, and liver weight (Supplemental Figure 7; first compared with third violin plot). However, fasting glucose (Figure 5B; first compared with third violin plot), systemic ALT levels (Figure 5E; first compared with third violin plot), and liver triglyceride accumulation (Figure 5F; first compared with third violin plot) were reduced in mice lacking PKM2 expression in adipocytes. Further, histological analyses of the liver revealed slight reduction in macrovesicular steatosis (Figure 5H; first compared with third panel) coupled with subtle difference in steatosis grade and inflammatory score (Figure 5H, first compared with third panel; and Table 1; 3 to 2/3, 90% to 80%–90%, and 1 [focal 2] to 0; first vs. second row of data). Last, the observed improvements in metabolic parameters correlated with a trend in reduced eWAT immune cell accrual in vehicle-treated PKM2-sufficient (WT + Pkm2^fl/fl^ Adipoq^Cre–^) compared with PKM2-deficient (Pkm2^fl/fl^ Adipoq^Cre+^) mice as determined by flow cytometry (Figure 5G; first compared with third violin plot) and histological analyses (Figure 5I, first compared with third panel). These data suggested that adipocyte PKM2 expression independent of body weight changes may contribute to metabolic disease severity in obesity.

Given the observed changes, therapeutic potential of PKM2 modulation on metabolic disease severity was examined next. Pkm2^fl/fl^ Adipoq sufficient (WT + Pkm2^fl/fl^ Adipoq^Cre–^) and Pkm2^fl/fl^ Adipoq deficient (Pkm2^fl/fl^ Adipoq^Cre+^) mice fed HFD were either vehicle-treated (DMSO; above) or treated with TEPP-46 (i.p. injection every 3 days for 2 weeks) in vivo (Figure 5A). TEPP-46 treatment regardless of mouse genotype did not impact body weight, adiposity, spleen weight, or liver weight (Supplemental Figure 7). Notably, TEPP-46 treatment of PKM2-sufficient (WT + Pkm2^fl/fl^ Adipoq^Cre–^) mice improved fasting glucose (Figure 5B; first compared with second violin plot), fasting insulin (Figure 5C; first compared with second violin plot), and the Homeostatic Model Assessment of Insulin Resistance (HOMA-IR) index value (Figure 5D; first compared with second violin plot) while showing a trend in reduction of systemic ALT levels (Figure 5E; first compared with second violin plot) and reduction in liver triglyceride accumulation (Figure 5F; first compared with second violin plot). Further, histological analyses of the liver revealed robust impact of TEPP-46 treatment on macrovesicular steatosis (Figure 5H; first compared with second panel) that was coupled with reduced steatosis grade and percent and inflammatory score (Figure 5H, first compared with second panel; and Table 1; 3 to 2, 90% to 55%, and 1 [focal 2] to 0; first vs. third row of data). Last, TEPP-46 treatment of PKM2-sufficient (WT + Pkm2^fl/fl^ Adipoq^Cre–^) mice, compared with vehicle-treated controls, yielded a trend in reduction of eWAT immune cell accrual as determined by flow cytometry (Figure 5G; first compared with second violin plot) and histological analyses (Figure 5I, first compared with second panel).

Importantly, compared with vehicle-treated PKM2-deficient (Pkm2^fl/fl^ Adipoq^Cre+^) mice, TEPP-46 treatment did not reveal additive improvement in fasting glucose (Figure 5B; third compared with fourth violin plot) and insulin levels (Figure 5C; third compared with fourth violin plot), HOMA-IR index value (Figure 5D; third compared with fourth violin plot), systemic ALT levels (Figure 5E; third compared with fourth violin plot), and hepatic triglyceride accumulation (Figure 5F; third compared with fourth violin plot). Similarly, histological analyses of the liver revealed that TEPP-46 treatment had limited impact on macrovesicular steatosis (Figure 5H; third compared with fourth panel) as well as steatosis grade and percent and inflammatory score (Figure 5H, third compared with fourth panel; and Table 1; 2/3 to 2/3, 80%–90% to 85%, and 0 to 0; second vs. fourth row of data) and on a reduction in eWAT immune cell accrual compared with vehicle-treated controls as determined by flow cytometry (Figure 5G; third compared with fourth violin plot) and histological analyses (Figure 5I, third compared with fourth panel). Together, these data suggested that TEPP-46 treatment in vivo may hold therapeutic promise in limiting severity of obesity-associated metabolic diseases and that the beneficial actions of TEPP-46 treatment in obese mice may in part depend on adipocyte PKM2 expression.

### IFN-I/PKM2 axis regulates inflammatory cytokine production by human white adipocytes.

**Figure 6:**
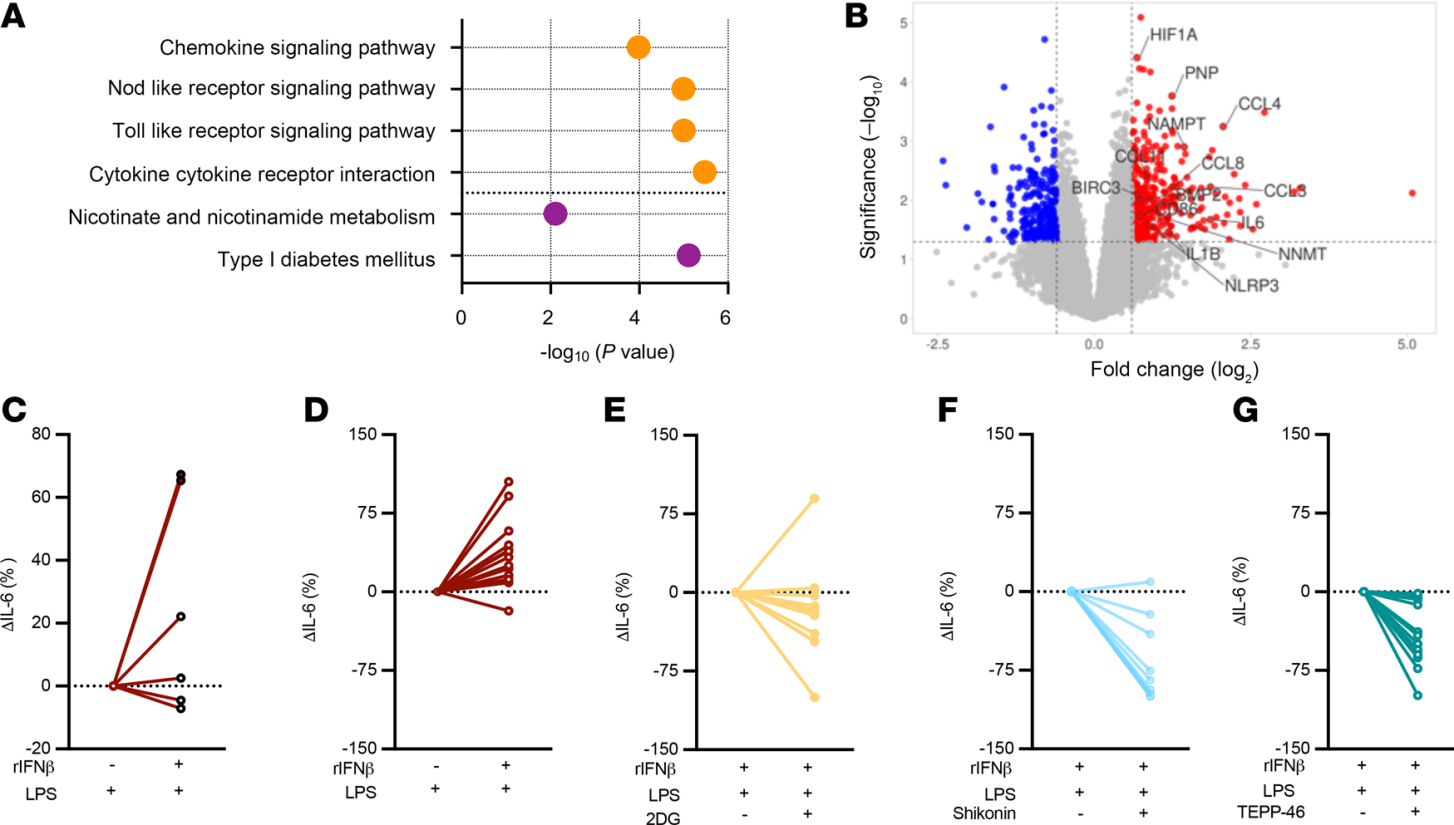
IFN-I/PKM2 axis regulates inflammatory cytokine production in human adipocytes. (**A** and **B**) Omental WAT biopsies were obtained from MH and MC cohorts, and transcriptome was analyzed via bulk RNA-Seq analyses. (**A**) ToppGene pathway enrichment analysis of differentially expressed genes between MH and MC cohorts. Pathways related to inflammation signaling (orange) and cellular metabolism (purple) are highlighted. (**B**) Volcano plot of gene expression levels of WAT between MH and MC cohorts. Genes significantly upregulated (fold change > 1.5, *P* value < 0.05) in MC cohort are represented by red dots. Genes significantly downregulated (fold change < –1.5, *P* value < 0.05) in MC cohort are represented by blue dots. Representative upregulated genes related to immune activation and cellular metabolism depicted in **A** are highlighted. (**C**) Primary adipocytes were isolated from omental WAT biopsies obtained from MH and MC cohort. Adipocytes were stimulated with vehicle control (saline) or rIFNβ (500 U) for 3 hours and subsequently challenged with LPS (100 ng/mL) for 4 hours. Culture supernatants were collected, and IL-6 levels were measured via ELISA. Data depict percentage change of IL-6 levels compared with vehicle + LPS–stimulated condition. (**D**–**G**) Adipocytes were derived from SVF isolated from omental WAT biopsies obtained from MH cohort. (**D**) SVF-derived adipocytes were stimulated with vehicle control (saline) or rIFNβ (500 U) for 3 hours and subsequently challenged with LPS (100 ng/mL) for 4 hours. Culture supernatants were collected, and IL-6 levels were measured via ELISA. Data depict percentage change of IL-6 levels compared with vehicle + LPS–stimulated condition. (**E**–**G**) SVF-derived adipocytes were stimulated with rIFNβ (500 U) in presence or absence of (**E**) 2-DG (2 mM), (**F**) shikonin (10 μM), or (**G**) TEPP-46 (100–200 μM) and were subsequently challenged with LPS (100 ng/mL) for 4 hours. Culture supernatants were collected, and IL-6 levels were measured via ELISA. Data depict percentage change of IL-6 levels compared with rIFNβ + LPS–stimulated condition.

Humans with obesity exhibit low-grade chronic inflammation, both systemically and in WAT. To begin to uncover the congruency of our mouse findings to human disease, we established a small human cohort of adolescents living with obesity and undergoing bariatric surgery. In this cohort, individuals living with obesity were stratified into 2 groups based clinical parameters of metabolic disease: metabolically healthy individuals (MH) and individuals with metabolic disease (MC) (Table 2). Despite a significant increase in HA1c in MC individuals, the systemic levels of inflammatory cytokines were similar between the groups (Table 2). These data suggest that the presence of metabolic diseases in these individuals may be linked dominantly with tissue instead of peripheral inflammation. To begin to understand potential differences in tissue inflammation, WAT obtained from MH and MC individuals was subjected to RNA-Seq analysis. Notably, WAT of MC individuals compared with MH individuals had marked increase in expression of both inflammatory and metabolic genes (Figure 6, A and B). Further, adipocytes from MC versus MH individuals when exposed to IFN-I + LPS produced higher levels of pro-inflammatory cytokines (12). Thus, whether the increase in WAT inflammation in MC individuals may be dependent on the activation of the IFN-I/PKM2 axis was examined next. White adipocytes were isolated from the omental WAT of individuals living with obesity (12, 52, 53). Notably, exposure of both primary (Figure 6C) and SVF-derived (Figure 6D) human white adipocytes to combined IFN-I + LPS resulted in increased pro-inflammatory cytokine production. Given our findings in mice, we next examined if the manipulation of the glycolytic pathway and PKM2 function would similarly impact human white adipocytes’ ability to produce pro-inflammatory cytokines. Notably, inhibition of glycolysis by either 2-DG or shikonin treatment abrogated human SVF-derived white adipocyte pro-inflammatory cytokine production (Figure 6, E and F). Further, modulation of PKM2 function by TEPP-46 treatment was also sufficient to reduce cytokine production by SVF-derived human white adipocytes (Figure 6G). Whether these outcomes were divergent in MC versus MH individuals was also studied. SVF-derived white adipocytes from MH and MC individuals showed a similar reduction in cytokine production after shikonin, 2-DG, or TEPP-46 treatment (Supplemental Figure 8, A–E). Last, we compared systemic and SVF white adipocyte–produced IL-6 levels between the 2 groups. Surprisingly, despite small cohort size, SVF-derived white adipocytes from MC individuals showed a trend toward higher IL-6 production compared with those isolated from MH counterparts (Supplemental Figure 8F). Together, these data suggest that the inflammatory function of the IFN-I/PKM2 axis is conserved in human white adipocytes and that MC adipocytes may be more inflammatory.

## Discussion

Here, we demonstrate that activation of the IFN-I/PKM2 axis may link dysregulated white adipocyte inflammatory vigor with severity of obesity-associated metabolic diseases. We show that IFN-I sensing, via IFNAR signaling, skews white adipocyte metabolism toward utilization of NAD^+^ and PKM2-dependent glycolysis to increase adipocyte inflammatory cytokine production. In contrast, using complementary chemical and genetic approaches, we display the necessity of the NAD^+^ salvage pathway, glycolysis, or PKM2 functions in restricting IFN-I–driven amplification of white adipocyte inflammatory cytokine production that is associated with reduction in severity of metabolic diseases in obese mice. Importantly, we also show that such processes are conserved in human white adipocytes obtained from individuals living with obesity.

We demonstrate a strong enrichment of inflammatory pathways in white adipocytes after combined exposure to IFN-β and LPS. Further, genetic deletion of IFNAR expression in white adipocytes was sufficient to restrict systemic inflammatory response to LPS in a manner comparable to deletion of IFNAR expression in immune cells. These data potentially implicate adipocyte IFNAR signaling with systemic inflammatory responses to stimuli such as LPS — a process potentially relevant to individuals living with obesity given they exhibit increased IFN-I (12) and systemic LPS (11) levels.

Mechanistically, our findings indicate that IFN-β acts as a cellular signal that skews PKM2 toward its monomer/dimer state in white adipocytes. PKM2 is a regulatory enzyme of glycolysis, and its activity/downstream effector function is regulated by its oligomeric state. Shift to the oligomeric/tetrameric state drives the conversion of PEP to pyruvate within glycolysis, while a shift to monomeric/dimeric state allows for its nuclear translocation and subsequent transcription of genes involved in metabolism and inflammation (31). Building on previous knowledge of PKM2 oligomeric state contributions to immune cell inflammatory/tumorigenic functions (45, 54), herein, we show how PKM2 function shapes white adipocyte pro-inflammatory cytokine production. Notably, IFN-I–driven shift toward PKM2 monomeric/dimeric state, and subsequent priming toward inflammatory gene expression, may explain its ability to amplify LPS-driven cytokine production in white adipocytes. Further, our data show that limiting PKM2 to its tetrameric state via TEPP-46 is sufficient to restrict IFN-I–driven, but not solely LPS-driven, amplification of inflammatory cytokine production by white adipocytes in vitro. Future work focused on elucidating additional signals impacting PKM2 state may improve our understanding of regulation of PKM2 function in white adipocytes and could lead to targeted therapies toward various diseases associated with dysregulated adipocyte inflammation.

We also examined the contribution of adipocyte-intrinsic PKM2 to obesity-associated metabolic disease severity in vivo. Obese mice with an adipocyte-specific *PKM2* deficiency showed reduced levels of fasting glucose, fasting insulin, HOMA-IR index value, and ALT. Notably, in obese mice with an adipocyte-specific *PKM2* deficiency, TEPP-46 treatment (which also impacts PKM2 expressed in nonadipocytes) did not further restrict disease parameters. In contrast with these findings, a previous report showed that germline loss of *PKM2* promotes metabolic syndrome and hepatocellular carcinoma (55). These results hint that adipocyte PKM2 may hold importance in metabolic disease pathogenesis.

Mechanisms underlying IFN-I–driven modulation of PKM2 function in white adipocytes remain unknown. IFN-I signals through the IFNAR. Signaling downstream of IFNAR can lead to canonical JAK/STAT or noncanonical JAK1/TYK2 activation. JAK/STAT signaling is associated with regulation of cellular metabolism and specifically glycolysis (56, 57). Whether canonical or noncanonical pathways of IFNAR signaling may regulate PKM2 state was studied. Inhibition of the canonical JAK/STAT pathway by ruxolitinib failed to impact PKM2 monomer/tetramer ratios in the context of IFN-I + LPS sensing (DNS). These data suggest that the noncanonical IFNAR signaling pathway may play a dominant role in shaping PKM2 function. In addition to surface receptor–driven modulation, posttranslational modifications (PTMs) including phosphorylation (58, 59) can induce shifts in PKM2 monomeric/dimeric over tetrameric state formation. Specifically, phosphorylation of PKM2 at multiple tyrosine residues (e.g., Tyr 83, Tyr105, Tyr148, Tyr175, Tyr370, and Tyr390) reduces tetramer formation (60, 61), and phosphorylation at Ser37 mediates entry into the nucleus (33). We found that exposure of adipocytes to IFN-I + LPS in the presence or absence of TEPP-46 did not modify total PKM2 phosphorylation at Tyr105 (DNS). These data suggest other PTM processes, including sumoylation, acetylation, or protein-protein interactions, may have a more dominant impact on PKM2 function in the context of IFN-I response (34, 35).

Inflammatory microenvironment triggers glycolysis to regenerate NAD^+^ to meet rapid increases in cellular demand to support a pro-inflammatory phenotype (62). Our findings show that NAD^+^ pathways may contribute to white adipocyte pro-inflammatory cytokine production. Specifically, inhibition of NAMPT, which regulates the first step in the biosynthesis of NAD^+^ from nicotinamide (63), was sufficient to reduce LPS-driven cytokine production (Figure 4D) and decreased PKM2 monomer-to-tetramer ratio in white adipocytes treated with IFN-I (DNS). These data link NAD^+^ with IFN-I sensing and PKM2 oligomerization status. In fact, in addition to acting as an important coenzyme for redox reactions, NAD^+^ also serves as a cofactor for sirtuins. Specifically, NAD^+^ is necessary for SIRT5 function. In turn, SIRT5 mediates desuccinylation of PKM2, inhibiting dimerization, thereby modulating PKM2 downstream effector functions (34, 64, 65). Thus, further studies investigating whether and how differential SIRT5 expression/function in the context of IFN-I–mediated NAD^+^ skewing impacts PKM2 oligomeric status and possibly white adipocyte pro-inflammatory cytokine production are warranted.

Last, we observed the conservation of inflammatory and metabolic pathways and associated gene expression between mouse and human white adipocytes. In fact, in both mouse and human white adipocytes, IFN-I sensing increased the expression of genes/pathways associated with TLR signaling, chemokine signaling (inflammatory pathways), type II diabetes, and nicotinamide metabolism. Further, inflammation in human white adipocytes was restricted upon inhibition of glycolysis. Thus, the conserved impact of white adipocyte cellular metabolism and adipocyte inflammation in both mice and humans invoke the potential relevance of the IFN-I/PKM2 axis in progression of obesity-associated metabolic and nonmetabolic diseases in humans. The increases in IL-6 levels are linked with metabolic disease severity in humans (66) Akin to immune cells, white adipocyte sensing of inflammatory environment may yield changes in the intracellular metabolism and trigger production of various cytokines/adipokines and chemokines, in turn promoting systemic and tissue inflammation (e.g., accrual of diverse immune cells in WAT). Evolutionarily, this could have served as a benefit on an organismal level to provide another layer of immune responsiveness. However, in obesity, increased production of white adipocyte–derived cytokines could play a detrimental role. Our data suggest a potential for increased dependency of white adipocytes from MC individuals on glycolysis and PKM2 function for IL-6 production compared with white adipocytes from MH individuals. These highly preliminary (due to small cohort size) but provocative findings suggest additional analyses of factors that differentiate white adipocyte function in healthy obese versus metabolically challenged individuals with obesity are warranted.

In conclusion, herein we report the importance of IFN-Is in skewing white adipocytes’ cellular metabolism, which in turn amplifies responsiveness to inflammatory stimulus–driven cytokine production. Mechanistically, our findings demonstrate that IFN-I preferentially skews PKM2 to its monomeric/dimeric state and modulates NAD^+^ intracellular levels to increase white adipocyte inflammatory responsiveness. Notably, we also show the IFN-I/PKM2 axis within adipocytes may contribute to severity of metabolic disease in obesity. These unexpected perspectives on mechanisms that drive white adipocyte inflammation may present a platform to design and investigate new therapeutic targets for dysregulated inflammation in obesity.

Our insights into the adipocyte IFN-I/PKM2 axis and its function in white adipocyte inflammatory phenotype provide the opportunity to establish or redirect available treatments to target dysregulated white adipocyte inflammation in obesity. Interestingly unlike 2-DG or TEPP-46, chemical inhibition of intermediate steps in the glycolytic pathway (e.g., inhibition of PFKFB3 via 3PO) failed to restrict LPS-driven cytokine production in IFN-I–treated white adipocytes. Given the complexity of cellular metabolic pathways, this observation could be explained by compensatory activation of complementary glycolytic intermediates feeding back into the glycolytic pathway, such as pentose phosphate, aldose acid, and polyol pathways (58, 67). Whether IFN-Is directly impact potential compensatory cellular metabolic pathways was not examined in our study. Our data also link IFN-Is and NAD^+^ with pro-inflammatory cytokine production in white adipocytes. However, we did not investigate whether such outcomes are dependent on IFNAR signaling or conserved in obese mice.

Our study employed the Adipoq-Cre mouse line to study the contribution of adipocyte PKM2 expression in adipocyte inflammatory capacity and metabolic disease severity. However, recent literature has hinted that adiponectin expression may not be adipocyte specific (68–70). Thus, potential contribution of other cell types should be considered when interpreting our data. Notably, WAT is diverse in size, location, and function depending on their bodily location (71). Similarly, white adipocytes exhibit high degree of plasticity and have unique functional abilities depending on the WAT depot they reside in (72, 73). Our study did not investigate the role of IFN-I/PKM2 axis in specific WAT depots or subsets of white adipocytes. Such approaches would be beneficial to potentially uncover unique microenvironmental cues sufficient to create site-specific white adipocyte inflammation. Last, although our human cohort data hinted at trends in directionality linking adipocyte IL-6 with clinical parameters of metabolic diseases, our cohort size is too small to make robust conclusions. These shortcomings suggest a caution with overinterpretation of our potentially novel findings and require future analyses to include increased cohort size for definitive validation.

## Methods

### Sex as a biological variable.

Our study examined mostly male animals. However, the baseline characteristic of induction of cytokine production by adipocytes exposed to IFN-I and LPS was recapitulated in female mice and in human samples.

### Mouse obesogenic diet model.

All mice used were males on a C57BL/6 background (Jackson Laboratory). WT, IFNAR^–/–^, Adipoq^Cre^ IFNAR^fl/fl^ (IFNAR^fl/fl^ Adipoq^Cre^), and Adipoq^Cre^ Pkm2^fl/fl^ (Pkm2^fl/fl^ Adipoq^Cre^) mice were bred at Cincinnati Children’s Hospital Medical Center (CCHMC) in a specific pathogen–free facility maintained at 22°C, with free access to autoclaved low-fat CD food (LAB Diet 5010; calories provided by carbohydrates [58%], fat [13%] and protein [29%]) and water. At 8 weeks of age, mice were fed either irradiated HFD (Research Diets D12492; 60% of calories from fat) or CD. Food was replenished on a weekly basis to avoid contamination. Weight gain was monitored on a weekly basis. Mice were fasted overnight prior to glucose metabolism testing, ALT testing, or terminal harvest.

### Mouse primary adipocytes.

iWAT tissue was isolated and digested (1 mg/mL collagenase type IV, dispase 2, CaCl_2_) as previously described (74). The SVF-containing preadipocytes were cultured until confluence and differentiated as previously described (74). Briefly, after reaching confluence, preadipocytes were subjected to initiation media (Growth media: DMEM:F12, 10% FBS, 1% Pen/Strep, 1% l-glut; rosiglitazone; dexamethasone; 3-Isobutyl-1-methylxanthine [IBMX]; insulin) for 2 days. Afterward, cells were switched to continuation media (Growth media, rosiglitazone, insulin) for 2 days, followed by differentiation media (Growth media, insulin) for an additional 2–4 days to reach maximal differentiation prior to performing the experiment. Finally, on the day of the experiment, differentiation media were removed, and cells were placed in 1 mL of Growth media: DMEM:F12, FBS, Pen/Strep, l-glut. To account for divergent baseline cytokine production by in vitro–cultured adipocytes observed regardless of genotype (DNS), the percentage change in cytokine production is used to depict all data presented in this manuscript.

### eWAT and liver immune infiltration isolation and analysis.

eWAT-infiltrating immune cells were isolated from obese WT, PKM2^fl/fl^ Adipoq^Cre–^, and PKM2^fl/fl^ Adipoq^Cre+^ mice as previously described (52, 53). Briefly, fat pads were dissected and cut into small pieces. Tissue was digested using Liberase (21 μg/mL, Roche) and DNase I (8.8 μg/mL, Roche) in DMEM containing HEPES and 2% BSA. Tissue was incubated at 37°C for 45 minutes at 220 rpm. After digestion cells were filtered through a 100 μM strainer and centrifuged at 800*g* for 5 minutes. Followed by red blood cell lysis, single-cell suspension was subsequently analyzed by flow cytometry.

### Mouse eWAT mature adipocyte isolation.

eWAT was isolated from obese WT mice. eWAT was processed as described in *eWAT and liver immune infiltration isolation and analysis*. However, after digestion and first filter using 100 μM filter, mature adipocytes were collected and cultured in growth media (DMEM:F12, FBS, Pen/Strep) for 24 hours. Mature adipocytes were treated with saline (NS) or IFN-β (250 U/mL) for 3 hours, followed by LPS (100 ng/mL) for 4 hours. Collection of supernatants was later utilized for measurement of cytokines by ELISA.

### Hepatic immune cell isolation.

Hepatic immune cells were isolated from obese WT and Pkm2^fl/fl^ Adipoq^Cre^ mice and processed as previously described (52, 53). Briefly, whole liver was homogenized using Miltenyi Biotec Gentlemax C tubes using RPMI + 10% FBS. After homogenization cells were centrifuged at 930*g* for 10 minutes. Cell pellets were homogenized in a 33% Percoll solution (Sigma-Aldrich) diluted in RPMI medium 1640 (Gibco). Following gradient separation, and lysing of red blood cells, hepatic immune cells were analyzed by flow cytometry.

### Flow cytometry.

To determine immune cell population single-cell suspensions derived from hepatic and adipose tissues, isolated immune cells were labeled with monoclonal antibodies. First, total single cells were stimulated for 4 hours with PMA (50 ng/mL; Sigma-Aldrich) and ionomycin (1 μg/mL; Calbiochem), in the presence of Brefeldin A (10 mg/mL; GoldBio). Subsequently, cells were stained with Live/Dead stain (Zombie UV Dye; BioLegend) and CD45-PE-Dazzle594 (BioLegend, clone 104), then fixed, using Cytofix/Cytoperm Fixation/Permeabilization Kit (BD Biosciences). Data were collected using an LSR Fortessa flow cytometer (BD Biosciences) and analyzed by FlowJo software (Tree Star).

### Mouse hepatic function and phenotyping.

Serum ALT levels were quantified using the ALT Reagent and Catatrol I and II (Catachem). Hepatic triglycerides were quantified using the Triglyceride Reagent and Triglyceride Standards (Pointe Scientific) as described previously (75–77) from WT and Pkm2^fl/fl^ Adipoq^Cre^ mice.

### In vivo cytokine quantification.

An in vivo cytokine capture assay was performed to quantify systemic IL-6 and TNF levels. Biotinylated capture antibodies against IL-6 (clone MP5-32C11; eBioscience) and TNF (clone TN3; eBioscience) were injected i.p., and 1 hour later, mice were exposed to i.p. injections of either saline (ctrl) or rIFNβ (10^4^ U). Then, 3 hours later, mice were subsequently challenged i.p. with LPS (25 μg), and 4 hours later (8 hours after initial injection of capture antibodies) terminal serum collection was performed (12, 76–78).

### Adipocyte cytokine quantification.

Murine WT primary adipocytes were cultured as described above. For stimulation studies, adipocytes were first treated with saline (NS), IFN-β (250 U/mL), IFN-β (250 U/mL) + 2-DG (2 mM), IFN-β (250 U/mL) + shikonin (10 μM), and 2-DG (2 mM) or shikonin (10 μM) for 3 hours. Subsequently, LPS (100 ng/mL), Pam2 (25 μg/mL), or Poly(I:C) (50 μg/mL) was added to the culture media for 4 hours. IL-6 and occasionally TNF was quantified by ELISA (BD Biosciences) per manufacturer’s instructions.

### Human participants.

Bariatric surgery participants were recruited. Exclusion criteria included alcohol abuse, viral and autoimmune hepatitis, and immunosuppression or steroid use. At time of surgery, adipose tissue was collected to isolate human SVF cells to culture them until they differentiated into mature SVF-derived adipocytes.

### Human primary adipocytes.

Isolation and digestion of omental WAT (40 mg/mL type II collagenase) collected at time of surgery were performed as previously described (12, 53). Briefly, digested tissue was filtered using 100 μM filter, centrifuged at 250*g*, and treated with ACK lysis buffer to isolate the SVF. SVF was cultured in expansion media (DMEM/F:12, 15% FBS, 1% Pen–strep) until confluence and then switched to human adipocyte differentiation media (DMEM/F:12, 1% Pen–strep, 2 mM glutamine, 15 mM HEPES, 10 mg/mL transferrin, 33 μM biotin, 0.5 μM insulin, 17 μM pantothenate, 0.1 μM dexamethasone, 2 nM T3, 500 μM IBMX, 1 μM ciglitazone) for 14–16 days. Next, cells were placed in human adipocyte maintenance media (DMEM/F:12, 1% Pen–strep, 2 mM glutamine, 15 mM HEPES, 10 mg/mL transferrin, 33 μM biotin, 0.5 μM insulin) for 7–10 days. Once fully differentiated, adipocytes were stimulated and processed for further analysis of inflammatory vigor.

### Human omental WAT mature adipocyte isolation.

Human omental WAT was processed as described above for primary adipocytes. Mature adipocytes were collected after digestion and filtration of WAT. Mature adipocytes were cultured in expansion media (DMEM:F12, 15% FBS, 1% Pen/Strep) for 24 hours and treated with saline (NS) and recombinant human IFN-β (500 U/mL) alone or together with metabolic inhibitors for 3 hours, followed by LPS (100 ng/mL) stimulation for 4 hours. Collection of supernatants was later utilized for measurement of cytokines by ELISA.

### Quantitative reverse transcription polymerase chain reaction.

Primary adipocytes were homogenized in TRIzol (Invitrogen) followed by RNA extraction, reverse transcription to cDNA (Verso cDNA Synthesis Kit; Thermo Fisher Scientific), and qPCR analysis (Light Cycler 480 II; Roche), according to manufacturer’s instruction as previously described.

The following primer pairs were used for mouse studies: *Ifnar1* For AGCAGGTAGAGAACTCGCCA Rev CTGCATCAGGAGGTGGAGTT – *Oas1a* For AGCAGGTAGAGAACTCGCCA Rev CTGCATCAGGAGGTGGAGTT – *Isg15* For GTCACGGACACCAGGAAATC Rev AAGCAGCCAGCCGCAGACTG – *Tlr4* For CATCCAGGAAGGCTTCCACA Rev GGCGATACAATTCCACCTGC.

The following primer pairs were used for human studies: *IFIT1* For AGTGGCTGATATCTGGGTGC Rev CCTCCTTGGGTTCGTCTACA – *OAS1* For AGCAGGTAGAGAACTCGCCA Rev CTGCATCAGGAGGTGGAGTT – *ISG15* For GAGAGGCAGCGAACTCATCT Rev CTTCAGCTCTGACACCGACA.

### Cellular bioenergetics quantification.

Primary adipocytes were plated at a concentration of 1 × 10^4^ cells per well in a polyethylenimine-precoated XF96 cell culture microplate (Agilent). Prior to bioenergetics analysis, adipocytes were treated with saline, IFN-β (250 U/mL), etomoxir (250 μM), and/or 2-DG (2 mM). An XF Analyzer (Seahorse Bioscience) was used to measure bioenergetics. Briefly, an XF96 extracellular flux assay cartridge (Seahorse Bioscience) was hydrated overnight at 37°C according to manufacturer’s instruction. XF assay medium supplemented with 25 mM glucose, 10 mM pyruvate, and 0.3% fatty–acid free BSA (pH 7.4) and incubated at 37°C in a non-CO_2_ incubator for 1 hour. Oligomycin (2 μg/mL), carbonyl cyanide *p*-trifluoromethoxyphenylhydrazone (FCCP; 1 μM), and rotenone (2.5 μM)/antimycin A (2.5 μM)/2-DG (10 mM) were sequentially injected and ECAR was quantified.

### Western blot analysis PKM2 oligomers.

Differentiated white adipocytes were stimulated with saline (NS), rIFNβ (250 U), LPS (100 ng/mL), or combination of rIFNβ (3 hours) + LPS (additional 4 hours) in the presence or absence of TEPP-46 (100–200 μM). Cells were then washed with PBS and stabilization of oligomers was performed by adding disuccinimidyl suberate (Thermo Fisher Scientific 21555) 1 mM for 30 minutes at room temperature (RT) followed by adding quench solution (10 mM Tris) for 15 minutes at RT. Next cells were lysed in RIPA buffer (Sigma 20188) with protease and phosphatase inhibitors (protease inhibitor — Roche 11836153001, phosphatase inhibitor — Thermo Fisher Scientific A32957). Total protein was quantified by Bradford protein assay and diluted to equivalent concentration of protein mixed with Laemmli+β-mercaptoethanol, then boiled 100°C for 5 minutes before loading onto gel. Total protein from each treatment group was separated using Mini-PROTEAN TGX polyacrylamide gels (Bio-Rad) and transferred to polyvinylidene difluoride membranes. Membranes were blocked for 1 hour and incubated overnight at 4°C with primary rabbit antibodies including α-tubulin (Cell Signaling Technology 2125) diluted in 5% milk/Tris-buffered saline with Tween 20 (TBST) (1:3,000), PKM2 (Cell Signaling Technology 4053) diluted in 5% milk/TBST (1:2,000). Thereafter, the membranes were incubated for 2 hours with specific horseradish peroxidase–conjugated secondary antibody (anti-rabbit IgG HRP, Cell Signaling Technology 7074, 1:5,000). Western blots were developed by enhanced chemiluminescence (PerkinElmer) per manufacturer instructions and detected by x-ray films.

### Bulk RNA-Seq and gene expression quantification.

RNA-Seq analysis was performed on visceral adipose tissue obtained during vertical sleeve gastrectomy surgery. Bulk RNA-Seq analysis was performed from total RNA following quantification (Qubit RNA assay kit) and library preparation (Illumina TrueSeq RNA preparation kit) by running 50 bp single-end reads (~20 million reads per sample). Gene expression analysis was performed using AltAnalyze (v2.1.1; (http://altanalyze.org). Single-end 50 bp reads were aligned to the human genome. Unsupervised analysis was performed using the software Iterative Clustering and Guide-gene Selection module within AltAnalyze. Differential gene expression was assessed using AltAnalyze’s built-in pipeline, which applies a moderated *t* test via the limma package on normalized expression data. Genes showing at least 1.5-fold and a *P* value < 0.05 were considered significantly differentially expressed. Pathway enrichment analysis was performed using ToppGene with both Reactome and KEGG pathways selected as reference databases.

### NMR-based metabolomics analysis.

Adipocyte SVF cultures were stimulated as described above and cells collected for NMR analyses. Briefly, media were removed, and plates were rinsed with cold PBS 2 times. Next cells were treated with a cold methanol/water mixture (80:20) for protein precipitation, then scraped, and the suspension was transferred to preweighted Eppendorf to pellet the protein and stored at –80°C until further use. The polar cell extracts, in 80% methanol, were dried in a SpeedVac centrifuge for 4–6 hours and stored at –20°C until further preparation for NMR data collection. On the day of data collection, the dried hydrophilic cell extract samples were resuspended in 220 μL of NMR buffer (100 mM potassium phosphate pH 7.3, 0.1% sodium azide, 1 mM trimethylsilylproprionate in 100% D_2_O). NMR experiments were conducted using 200 μL samples in 103.5 mm × 3 mm NMR tubes (Bruker). One-dimensional ^1^H NMR spectra were acquired on a Bruker Avance III HD 600 MHz spectrometer with prodigy BBO probe. Metabolites found in cell extract were assigned based on 1D ^1^H and 2D NMR experiments and reference spectra found in databases, such as the Human Metabolome Database, the Madison metabolomics consortium database, the biological magnetic resonance data bank, and Chenomx NMR Suite profiling software (version 8.1). The concentrations of the metabolites were calculated using Chenomx software based on internal standard, TMSP. The concentrations were normalized to total NMR spectral intensity from each sample to correct for differences in number of cells per sample prior to the statistical analyses.

### Histopathological analysis.

For all mice, identical positioned part of the liver was collected for histology. Liver tissue was fixed in 10% buffered formalin and stained with H&E. Specimens received in clinical pathology were accessioned with a unique identifier using a combination of EPIC Beaker and the Roche Vantage barcoding/tracking system that carries throughout the entire tissue process. Specimens were grossed, processed, and embedded in paraffin or OCT (for cryosectioning). Slides were sectioned to specifications of required stains. An H&E stain was routinely run on all specimens for basic morphology. More complex staining was available at the pathologist’s request. Slides for special staining, ISH, and IHC were run primarily through automation (except for a few select special stains) using the Roche/Ventana platforms: HE600, BenchMark Special Stainers, BenchMark Ultra XT, and Discovery XT. The slides were deparaffinized, pretreated, and stained. Coverslipping of stained slides was performed offline using the Epredia ClearVue cover-slipper. The glass histological slides were scanned using a Leica Aperio AT2 scanner. The scanner was set to scan at 40× magnification with a resolution of 0.25 μm/pixel using a 2× optical magnification changer on a 20×/0.75 NA Plan Apo objective lens. Evaluation was performed by an experienced board-certified pathologist blinded to experimental parameters while scoring according to standard practice (75, 76, 79).

### Statistics.

Statistical tests were utilized for all data sets with similar variance. For all normally distributed data unpaired 2-tailed Student’s *t* test was used to determine differences between 2 groups. For comparison of multiple groups ordinary 1-way ANOVA with comparison of multiple groups was performed. All data presented as means ± SEM. *P* < 0.05 was considered statistically significant. Analysis was performed via GraphPad Prism Software. Determined sample sizes were based on preliminary data with respect to obesity modeling including weight gain, immune cell infiltration, severity of obesity-associated metabolic sequelae, and interrogation of myeloid cell inflammatory vigor. No animals were excluded from analyses, and none of the studies were conducted in a blinded fashion.

### Study approval.

For mouse studies all care was provided in accordance with the *Guide for the Care and Use of Laboratory Animals* (National Academies Press, 2011) . All studies were approved by CCHMC IACUC. For human studies/samples written informed consent was obtained from participants from CCHMC Pediatric Diabetes and Obesity Center. Further recruitment and study protocols were approved by institutional review board at CCHMC, and studies were carried out in accordance with these guidelines.

### Data availability.

Raw data for the article are also available in the Supporting Data Values file. All RNA-Seq data sets are deposited in GEO and are publicly available: GSE110236 (mouse RNA-Seq) and GSE306518 (human RNA-Seq).

## Author contributions

MSMAD, PCA, CCC, TES, JRD, JRO, JLW, JE, HC, KS, CJU, MWC, LRR, JSG, RM, MAH, MEMF, and SD participated in data generation. MSMAD, PCA, CCC, SS, MEMF, and SD participated in analysis and interpretation of data. MSMAD and SD participated in the conception and design of the study and wrote the manuscript. All authors have reviewed the manuscript and approve the final version.

## Funding support

This work is the result of NIH funding, in whole or in part, and is subject to the NIH Public Access Policy. Through acceptance of this federal funding, the NIH has been given a right to make the work publicly available in PubMed Central.

- NIH R01DK099222, the CCHMC Pediatric Diabetes and Obesity Center (to SD and MAH).
- American Diabetes Association 1-18-IBS-100 (to SD).
- Department of Defense W81XWH2010392 (to SD and MAH).
- NIH R01DK099222-02S1 (associated with SD, MEMF, and JRO).
- CCRF Endowed Scholar Award (to SD).
- NIH T32AI118697 (associated with CCC).
- NIH T32GM063483-14 (associated with CCC).
- The Arnold W. Strauss Fellow Award (to JRD).
- NIH P30 DK078392 of the Digestive Disease Research Core Center at CCHMC (associated with SD).
- NIH P30 AR070549 of the Rheumatic Disease Resource Center in Cincinnati (associated with SD).
- JSG: American Heart Association (18CDA34080527).
- JSG: NIH (R21OD031907).
- JSG: CCHMC Trustee Award.
- JSG: CCHMC Center for Pediatric Genomics Award.
- JSG: CCHMC Center for Mendelian Genomics & Therapeutics Award.
- RM: American Heart Association Postdoctoral grant (19POST34380545).

## Supplementary Material

Supplemental data

Unedited blot and gel images

Supporting data values

## Figures and Tables

**Table 1:**
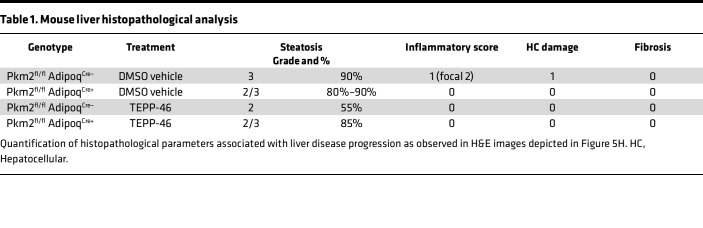
Mouse liver histopathological analysis

**Table 2:**
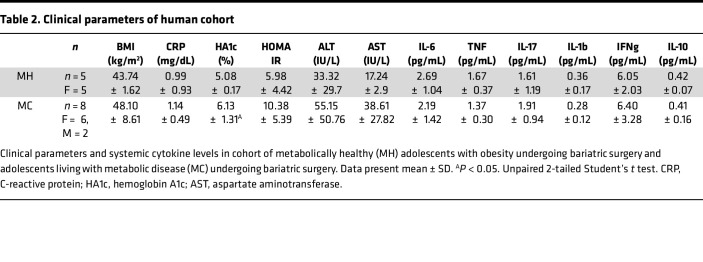
Clinical parameters of human cohort

## Version 1 11/24/2025

Electronic publication
