# Supplementary material for: A shift in PKM2 oligomeric state instructs adipocyte inflammatory potential
Source: JCI Insight. 2025 Nov 24;10(22):e185914. doi: 10.1172/jci.insight.185914 (PMC12643483; doi:10.1172/jci.insight.185914)
Supplement: Supplemental data [file jciinsight-10-185914-s100.pdf]

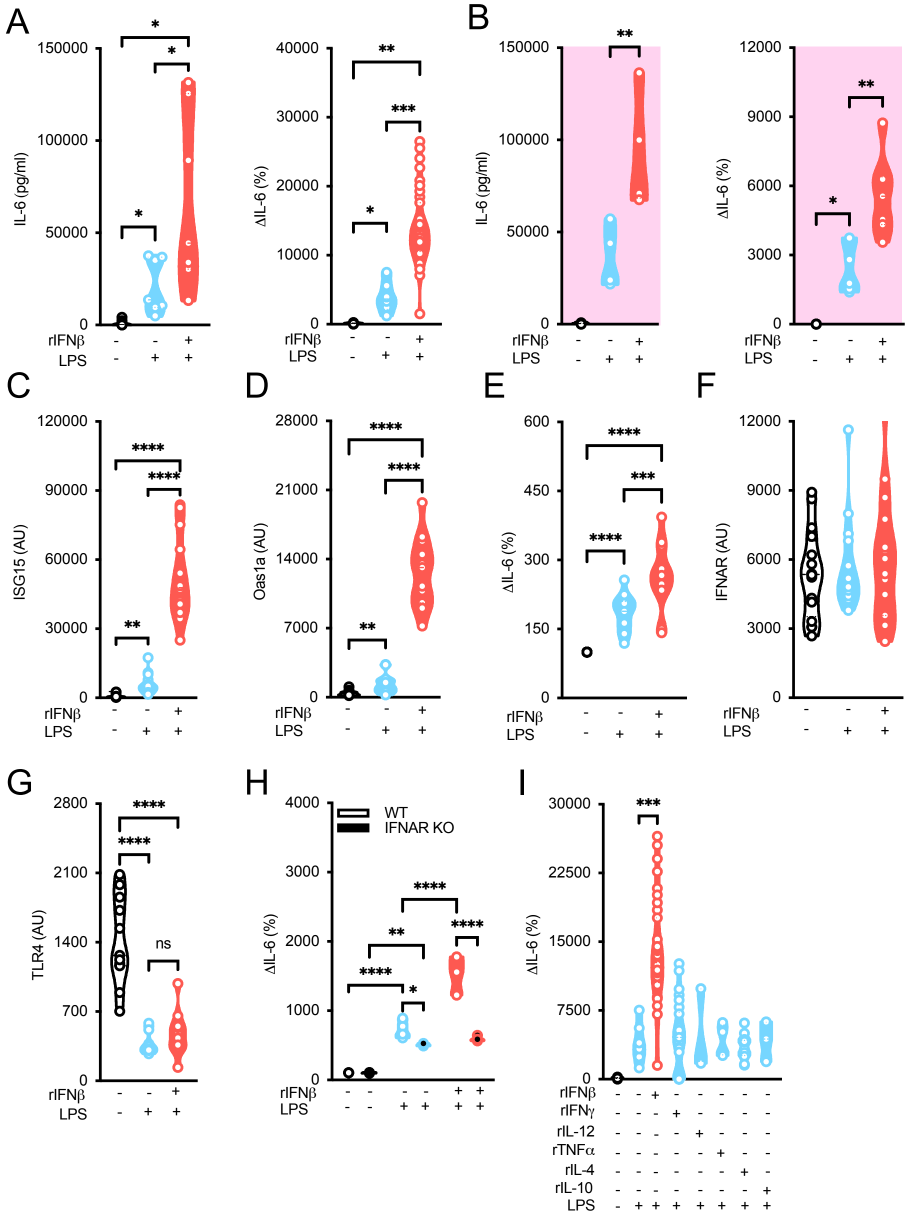

Supplemental Figure 1

Supplemental Figure 1. IFN-I/IFNAR axis uniquely amplifies adipocyte inflammatory vigor. (A-G) WT mouse adipocytes (SVF derived) were stimulated with vehicle control (saline) or rIFNβ (250 U) for 3 hours and subsequently challenged with LPS (100 ng/mL) or not for 4 hours. Culture supernatants were collected, and IL-6 levels (via ELISA) and gene expression (via qPCR) were measured. (A) Raw (left panel) and percent change of IL-6 (right panel) cytokine production in male adipocytes compared to vehicle-stimulated control. (B) Raw (left panel, pink shade) and percent change of IL-6 (right panel, pink shade) cytokine production in female adipocytes compared to vehicle-stimulated control. (C-D) Gene expression of *Isg15* (C), *Oas1a* (D) in adipocytes isolated from male mice. (E) Primary adipocytes isolated from iWAT of male mice were stimulated with vehicle control (saline) or rIFNβ (250 U) for 3 hours and subsequently challenged with LPS (100 ng/mL) or not for 4 hours. Culture supernatants were collected, and IL-6 levels were measured by ELISA. Percent change of IL-6 levels compared to vehicle-stimulated condition. (F, G) Gene expression of *Ifnar* (F), *Tlr4* (G) in adipocytes isolated from male WT mice. (H) WT and IFNAR KO mouse SVF-derived adipocytes were stimulated with vehicle control (saline) or rIFNβ (250 U) for 3 hours and subsequently challenged with LPS (100 ng/mL) or not for 4 hours. Percent change of IL-6 levels compared to vehicle-stimulated condition. (I) WT mouse adipocytes (SVF derived) were stimulated with vehicle control (saline) or rIFNβ (250 U; red), rIFNγ (20 ng/mL), rIL-12 (20 ng/mL), rTNF (20 ng/mL), rIL-4 (20 ng/mL), rIL-10 (20 ng/mL) for 3 hours and subsequently challenged with LPS (100 ng/mL) for 4 hours. Culture supernatants were collected, and IL-6 levels were measured via ELISA. Data depicts percent change of IL-6 levels compared to vehicle-stimulated controls. (A-H) In violin plots, data presents mean + SEM. Unless indicated no significance was observed. One-way ANOVA. *: $p < 0.05$; **: $p < 0.005$; ***: $p < 0.0005$; ****: $p < 0.00001$.

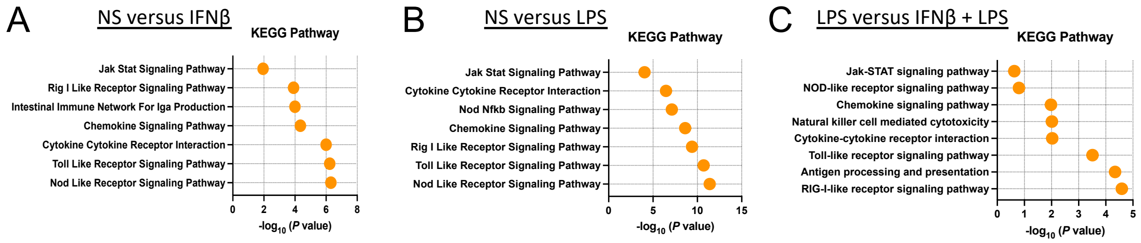

Supplemental Figure 2

Supplemental Figure 2. IFN-I sensing enhances inflammatory pathway activation in adipocytes. The data presented is related to Figure 1. WT mouse (SVF derived) adipocytes were stimulated with vehicle control (saline) or rIFNβ (250 U) for 3 hours and subsequently challenged with LPS (100 ng/mL) or not for 4 hours. ToppGene pathway enrichment analysis of upregulated differentially expressed genes (fold change > 2; p-value < 0.05) in rIFNβ-stimulated adipocytes compared to vehicle stimulated controls (A), in LPS-stimulated adipocytes compared to vehicle stimulated controls (B), and in rIFNβ + LPS-stimulated adipocytes compared to LPS-stimulated counterparts (C). Pathways related to inflammation are highlighted.

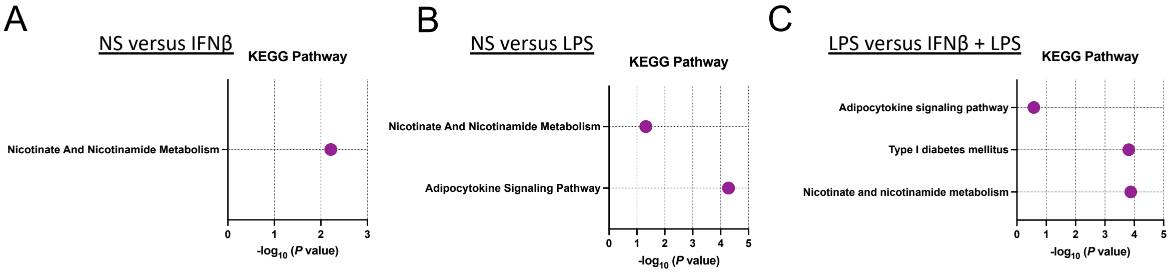

Supplemental Figure 3

Supplemental Figure 3. IFN-I sensing dominantly cellular metabolism pathways in adipocytes. The data presented is related to Figure 2. WT mouse (SVF derived) adipocytes were stimulated with vehicle control (saline) or rIFNβ (250 U) for 3 hours and subsequently challenged with LPS (100 ng/mL) or not for 4 hours. ToppGene pathway enrichment analysis of upregulated differentially expressed genes (fold change > 2; p-value < 0.05) in rIFNβ-stimulated adipocytes compared to vehicle stimulated controls (A), in LPS-stimulated adipocytes compared to vehicle stimulated controls (B), and in rIFNβ + LPS-stimulated adipocytes compared to LPS-stimulated counterparts (C). Pathways related to cellular metabolism are highlighted.

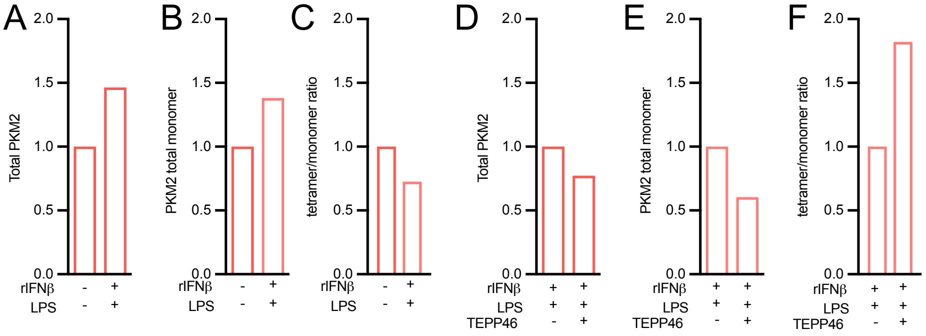

Supplemental Figure 4

Supplemental Figure 4. IFN-I and TEPP-46 modulate PKM2 state. The data presented is related to Figure 3. WT mouse (SVF derived) adipocytes were stimulated with vehicle control (saline) or rIFNβ (250 U) for 3 hours and subsequently challenged with LPS (100 ng/mL) or not for 4 hours. Cell lysates were collected, and protein expression of different configurations (monomer and tetramer) of PKM2 and α-tubulin (loading control) were analyzed via western blot. (A-C) Quantification of total (A), monomeric (B), and tetramer/monomer ratio (C) of PKM2 levels in rIFNβ + LPS-stimulated adipocytes relative to vehicle stimulated controls. (D-F) WT mouse (SVF derived) adipocytes were stimulated with rIFNβ + LPS in presence or absence of TEPP-46 (100 μM). Quantification of total (D), monomeric (E), and tetramer/monomer ratio (F) of PKM2 levels in rIFNβ + LPS + TEPP46 stimulated adipocytes relative to rIFNβ + LPS stimulated controls. (A-F) Representative of two independent experiments.

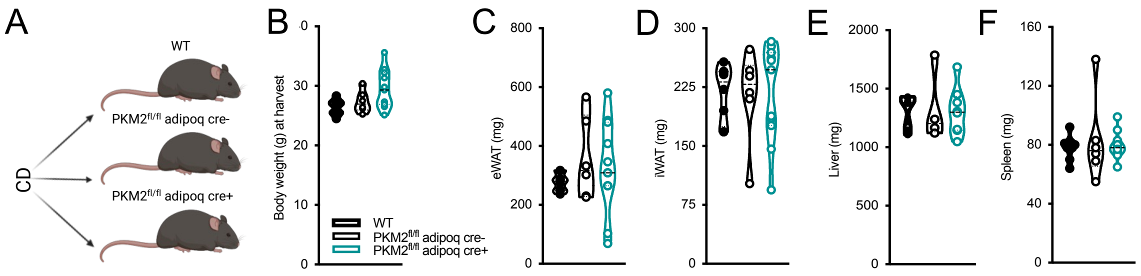

Supplemental Figure 5

Supplemental Figure 5. Adipocyte PKM2 expression does not alter body and tissue weight in lean mice. The data presented is related to Figure 5. WT, PKM2$^{fl/fl}$-Adipoq^{Cre-} , and PKM2$^{fl/fl}$-Adipoq^{Cre+} mice were fed chow diet (CD) for 34 weeks. (A) Schematic overview. (B) Body weight. (C) eWAT weight. (D) iWAT weight. (E) Spleen weight. (F) Liver weight. (B-F) In violin plots, data presents mean + SEM. Unless indicated no significance was observed. Unpaired Student's t-test.

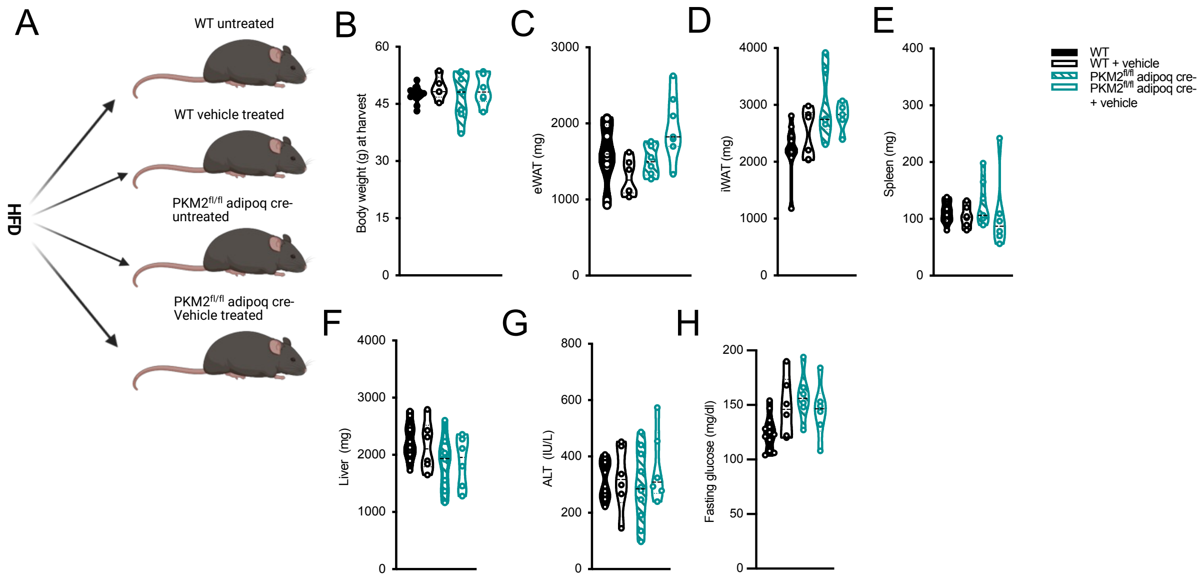

Supplemental Figure 6

Supplemental Figure 6. Adipocyte PKM2 expression or vehicle treatment does not alter body and tissue weight in obese mice. The data presented is related to Figure 5. Untreated WT, Vehicle treated WT, untreated PKM2$^{fl/fl}$-Adipoq^{Cre-} and vehicle treated PKM2$^{fl/fl}$-Adipoq^{Cre-} mice were fed high-fat diet (HFD) for 34 weeks. (A) Schematic overview. (B) Body weight. (C) eWAT weight. (D) iWAT weight. (E) Spleen weight. (F) Liver weight. (G) Liver triglyceride levels. (H) Serum ALT levels. (I) Fasting glucose levels. (B-I) In violin plots, data presents mean + SEM. Unless indicated no significance was observed.

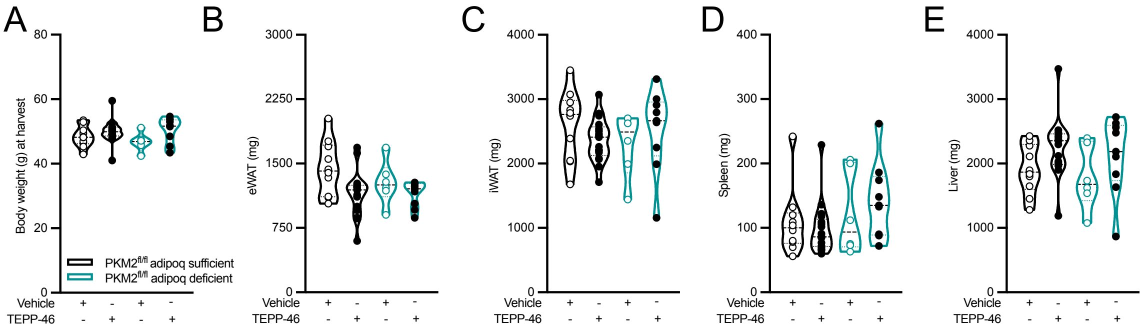

Supplemental Figure 7

Supplemental Figure 7. TEPP-46 treatment does not alter body and tissue weight in obese mice. The data presented is related to Figure 5. PKM2$^{fl/fl}$-Adipoq^{Cre-} and PKM2$^{fl/fl}$-Adipoq^{Cre+} mice were fed high-fat diet (HFD). After 34 weeks, mice were treated i.p. with vehicle control (DMSO) or TEPP-46 (37.5 mg/kg) every 3 days for 2 weeks. (A) Body weight. (B) eWAT weight. (C) iWAT weight. (D) Spleen weight. (E) Liver weight. (A-E) In violin plots, data presents mean + SEM. Unless indicated no significance was observed. One-way ANOVA.

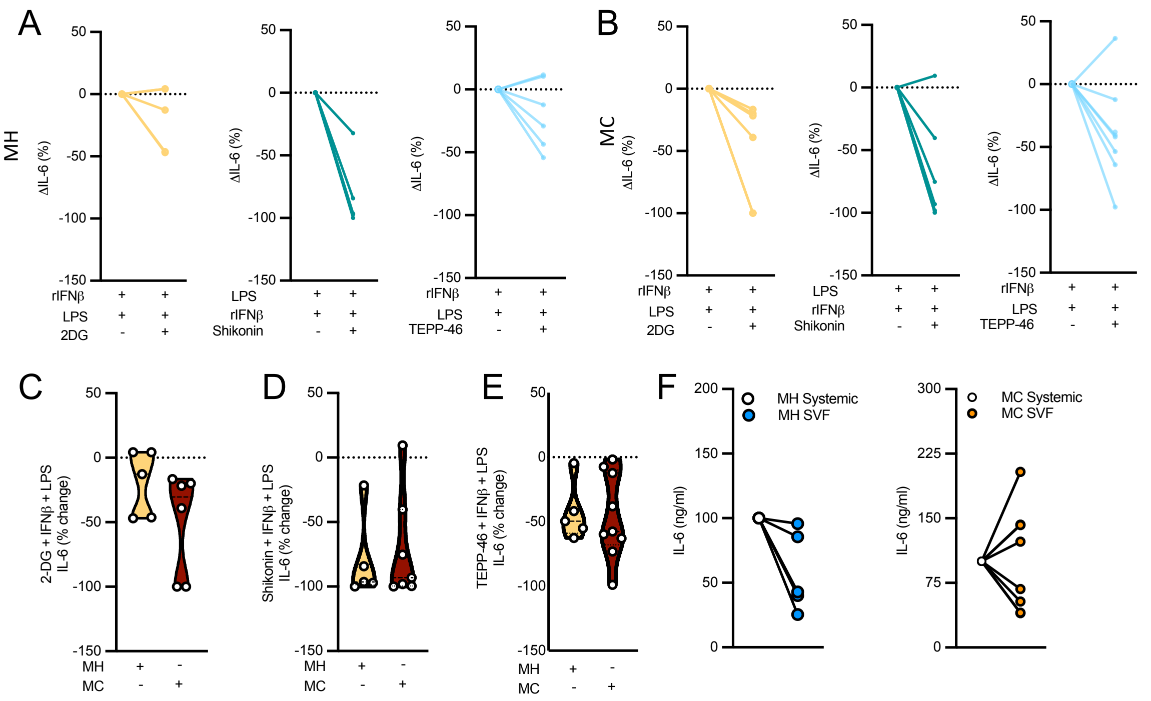

Supplemental Figure 8

Supplemental Figure 8. Comparison of IL-6 production in SVF derived white adipocytes from MH vs MC individuals. The data presented is related to Figure 6. Adipocytes were derived from SVF isolated from omental WAT biopsies obtained from MH and MC cohorts. (A-E) SVF-derived adipocytes were stimulated with rIFNβ (500 U) for 3 hours in presence or absence of 2-DG (2 mM), shikonin (10μM) or TEPP-46 (100-200 μM) and subsequently challenged with LPS (100 ng/mL) for 4 hours. Culture supernatants were collected, and IL-6 levels were measured via ELISA. (A, B) Percent change of IL-6 levels compared to rIFNβ + LPS-stimulated condition in adipocytes from MH (A) or MC cohorts (B). (C-E) Percent change of IL-6 levels in adipocytes from MH and MC cohorts stimulated with rIFNβ + LPS + 2-DG (C), rIFNβ + LPS + shikonin (D), or rIFNβ + LPS + TEPP-46 (E) compared to rIFNβ + LPS stimulated condition. (F) Comparison of IL-6 serum and SVF-derived adipocyte (unstimulated) levels by ELISA in MH and MC individuals. (B-D) In violin plots, data presents mean + SEM.
