# Supplementary figures and images for: A shift in PKM2 oligomeric state instructs adipocyte inflammatory potential
Source: JCI Insight. 2025 Nov 24;10(22):e185914. doi: 10.1172/jci.insight.185914 (PMC12643483; doi:10.1172/jci.insight.185914)

Figure 3D original blot PKM2

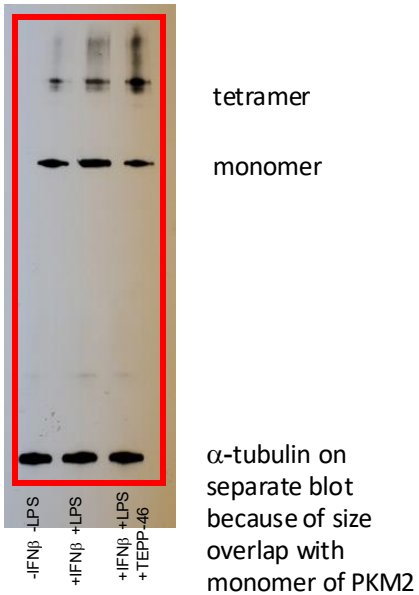

Supplement: Unedited blot and gel images [file jciinsight-10-185914-s101.pdf]
